# Rapamycin improves satellite cells’ autophagy and muscle regeneration during hypercapnia

**DOI:** 10.1172/jci.insight.182842

**Published:** 2025-01-09

**Authors:** Joseph Balnis, Emily L. Jackson, Lisa A. Drake, Diane V. Singer, Ramon Bossardi Ramos, Harold A. Singer, Ariel Jaitovich

**Affiliations:** 1 Division of Pulmonary and Critical Care Medicine and; 2 Department of Molecular and Cellular Physiology, Albany Medical College, Albany, New York, USA.

**Keywords:** Pulmonology, Autophagy, Mouse stem cells, Respiration

## Abstract

Both CO_2_ retention, or hypercapnia, and skeletal muscle dysfunction predict higher mortality in critically ill patients. Mechanistically, muscle injury and reduced myogenesis contribute to critical illness myopathy, and while hypercapnia causes muscle wasting, no research has been conducted on hypercapnia-driven dysfunctional myogenesis in vivo. Autophagy flux regulates myogenesis by supporting skeletal muscle stem cell — satellite cell — activation, and previous data suggest that hypercapnia inhibits autophagy. We tested whether hypercapnia worsens satellite cell autophagy flux and myogenic potential and if autophagy induction reverses these deficits. Satellite cell transplantation and lineage-tracing experiments showed that hypercapnia undermined satellite cells’ activation, replication, and myogenic capacity. Bulk and single-cell sequencing analyses indicated that hypercapnia disrupts autophagy, senescence, and other satellite cell programs. Autophagy activation was reduced in hypercapnic cultured myoblasts, and autophagy genetic knockdown phenocopied these changes in vitro. Rapamycin stimulation led to AMPK activation and downregulation of the mTOR pathway, which are both associated with accelerated autophagy flux and cell replication. Moreover, hypercapnic mice receiving rapamycin showed improved satellite cell autophagy flux, activation, replication rate, and posttransplantation myogenic capacity. In conclusion, we have shown that hypercapnia interferes with satellite cell activation, autophagy flux, and myogenesis, and systemic rapamycin administration improves these outcomes.

## Abstract 2

Hypercapnia and muscle wasting correlate with higher mortality in critical illness. Hypercapnia reduces stem cell activation, autophagy, and muscle regeneration. Rapamycin administration attenuate these deficits.

## Graphical abstract

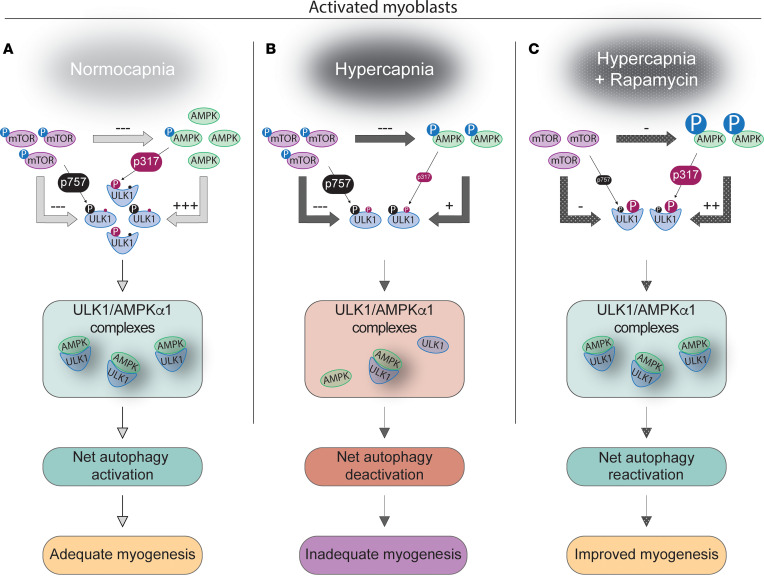

## Introduction

Critical illness–driven skeletal muscle dysfunction is independently associated with higher mortality and disability after hospital discharge (1, 2). CO_2_ retention, or hypercapnia, occurs in many critically ill patients receiving lung-protective protocols of mechanical ventilation (3). While these ventilatory strategies are beneficial for many patients (4), the development of hypercapnia predicts higher mortality as well (5). Previous research has indicated that hypercapnia leads to skeletal muscle dysfunction via accelerated protein catabolism and, simultaneously, attenuated protein synthesis (6, 7). While these processes take place in terminally differentiated myofibers, recent evidence also indicates that abnormal function of skeletal muscle stem cells, or satellite cells (8), can contribute to critical illness–driven muscle dysfunction (9). Importantly, biopsies obtained from critically ill patients revealed evidence of muscle injury, including disrupted sarcolemma integrity and macrophage infiltration (10). Concurrently, patients recovering from critical illness demonstrate a significant reduction of satellite cells, which reflects decreased regenerative potential (11). Recent animal data have shown that while satellite cells are required for the recovery of muscle integrity after critical illness, their capacity to engage in muscle regeneration is substantially reduced in that setting (12, 13). Importantly, adult satellite cells remain at the myofiber sublaminar area in a nonreplicative, or quiescent, state (8). Injurious events, such as critical illness, reactivate satellite cells (14). These activated cells reenter the cell cycle and support muscle regeneration through fusion with each other and with previously damaged myofibers (15). Even though elevated CO_2_ reduces cellular proliferation in multiple tissues (16, 17), very little research has focused on myogenesis in hypercapnia (18). Minimal research has been conducted on hypercapnia-driven dysfunctional satellite cells in vivo.

Autophagy is a cellular stress response pathway that orchestrates the removal of dysfunctional proteins and organelles, which are incorporated into vesicles and delivered to lysosomes for degradation (19). Autophagy is a key regulator of satellite cell activation and myogenesis (20). Indeed, autophagy flux supports the bioenergetic demands associated with satellite cells’ transition into the replicative state (21). Autophagy contributes to satellite cell activation at a critical time frame, when the cell responds to replicative stimulations, such as muscle injury. The loss of autophagy flux at that specific time point undermines satellite cells’ myogenic capacity (22). Autophagy also maintains satellite cells’ proteostatic properties that prevent their transition into cellular senescence (23). Importantly, while previous evidence indicates that hypercapnia inhibits autophagy flux in inflammatory cells (24), the interaction of hypercapnia, autophagy, and myogenesis has never been mechanistically investigated to our knowledge. Such research could accelerate the development of new strategies targeting muscle dysfunction in hypercapnia, with potential survival benefits for critically ill individuals.

Autophagy is canonically inhibited by the mechanistic target of rapamycin (mTOR) (25), which regulates multiple steps of autophagy, including induction (26, 27), nucleation (28) and autophagosome elongation (29), and maturation (30). By contrast, inhibition of mTOR by substrate shortage leads to autophagosome formation via AMP kinase (AMPK). AMPK phosphorylates unc-51 like autophagy activating kinase 1 (ULK1) (26, 27), which is an early event in autophagy activation. In the present work, we hypothesized that hypercapnia would undermine myogenesis because of a reduced autophagy activation in satellite cells, leading to impaired replication capacity. We also hypothesized that the mTOR pathway could be targeted to attenuate that deficit. Part of this study has been previously presented in an abstract form (31).

## Results

### Chronic hypercapnia exposure causes skeletal muscle dysfunction.

**Figure 1:**
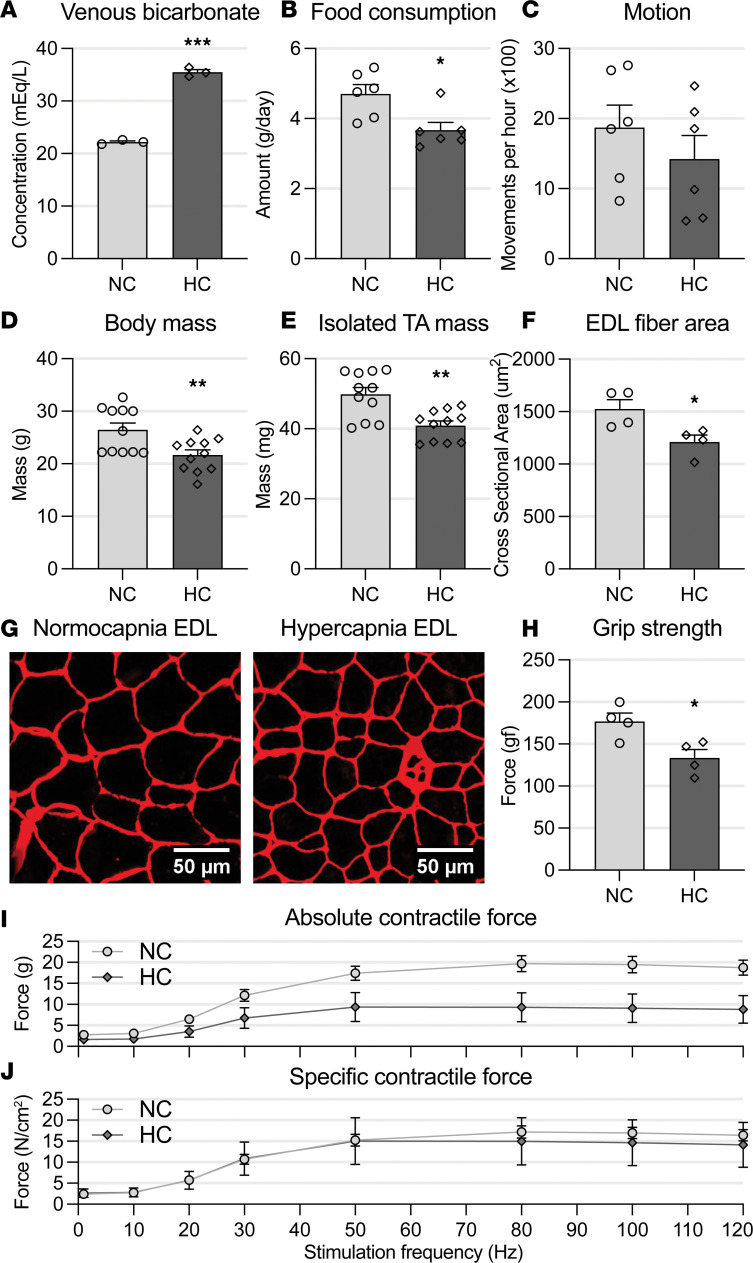
Chronic hypercapnia exposure causes skeletal muscle dysfunction. (**A**) Venous bicarbonate, a surrogate of chronic hypercapnia exposure, is elevated in the hypercapnic (HC) mice compared with their normocapnic (NC) counterparts (*n* = 3). (**B**) Food intake is significantly reduced in HC mice (*n* = 6). (**C**) Daily motion is not different between NC and HC mice (*n* = 6). (**D**) Gross body mass is reduced in HC mice in comparison with NC counterparts (*n* = 10 NC, 11 HC). (**E**) Freshly isolated tibialis anterior (TA) muscles from HC mice weigh less than NC mice–procured muscles (*n* = 11). (**F** and **G**) Sectioning and immunofluorescence stain of the extensor digitorum longus (EDL) muscle, automatically quantified, show reduced average myofiber cross-sectional area in HC mice (*n* = 4). (**H**) Mice in HC conditions have reduced limb grip strength (*n* = 4). (**I**) Isolated muscle contractility of EDL from HC mice shows lower absolute force when electrically stimulated in comparison with NC counterparts (*n* = 5 NC, 4 HC). (**J**) When corrected for muscle mass, HC muscles show no myofiber-specific force reduction when compared with NC littermates (*n* = 5 NC, 4 HC). All statistical comparisons were performed using Student’s *t* test; *P < 0.05, **P < 0.01, and ***P < 0.001.

Skeletal muscle dysfunction is caused by reduced muscle mass, force generation capacity, and regenerative potential (32–37). To investigate the effects of chronic hypercapnia on skeletal muscle, we housed 18- to 20-week-old C57BL/6 (C57) mice in a chamber that received a controlled gas mixture of 21% O_2_, 10% CO_2_, and 69% nitrogen. That environment has been shown by us (6) and others (38) to result in chronic normoxemic hypercapnia. Littermates kept in room air served as experimental controls. Consistently, determination of serum bicarbonate, which is a surrogate of chronic CO_2_ retention, was significantly elevated in hypercapnic mice (Figure 1A). As reduced food intake is known to upregulate autophagy flux and to contribute to muscle atrophy (39), we quantified food consumption and found it was significantly reduced in hypercapnic mice in comparison with normocapnic counterparts (Figure 1B). Unbiasedly recorded animal motion was not significantly different between conditions (Figure 1C), indicating that lower mobility is unlikely to confound the muscle atrophy effects of hypercapnia. As muscle wasting is characterized by reduced body weight (40), we weighed both normo- and hypercapnic mice and found that hypercapnia exposure led to reduced body mass (Figure 1D). To determine the effects of chronic CO_2_ elevation specifically on muscle mass, we weighed tibialis anterior (TA) muscles and found significantly reduced masses in animals exposed to hypercapnia compared with normocapnic controls (Figure 1E). Moreover, extensor digitorum longus (EDL) muscles were isolated and individual myofiber cross-sectional area was measured, revealing a reduced size of myofibers from hypercapnic animals (Figure 1, F and G). As most muscle atrophy models evoke a decreased force generation capacity, we conducted the grip strength test (6), which showed a reduced force generation in hypercapnic versus normocapnic mice (Figure 1H). Finally, ex vivo isolated contractility assays were performed (36), where EDL muscles collected from chronically hypercapnic mice showed a significant decrease in absolute, but not in specific, force relative to EDL muscles from normocapnic counterparts. These data indicate that the decrease in force generation capacity is due to a lessened muscle mass and not an intrinsic contractile deficit (Figure 1, I and J).

### Chronic hypercapnia undermines myogenesis.

**Figure 2:**
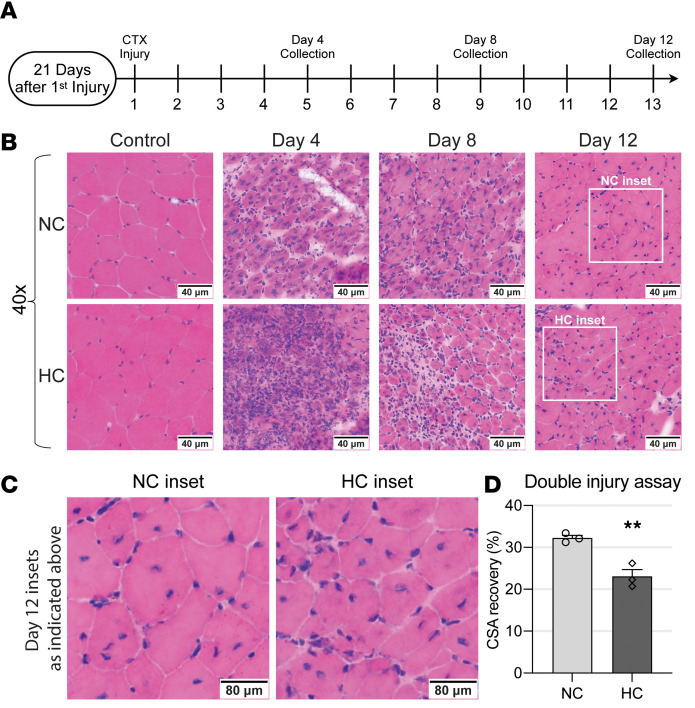
Chronic hypercapnia undermines myogenesis. (**A**) Double cardiotoxin (CTX) TA muscle injury timeline with various collection time points. (**B**) TA muscles from NC and HC animals were collected at 4, 8, and 12 days after the second cardiotoxin injury and were analyzed by H&E staining (*n* = 3). (**C** and **D**) Myofiber diameter after recovery remained significantly lower in HC mice 12 days after the second injury compared to contralateral control legs (*n* = 3). Statistical comparisons were performed using Student’s *t* test in **D**; **P < 0.01.

To investigate the effect of chronic hypercapnia on myogenesis, we conducted a systematic evaluation of the muscle recovery trajectory following cardiotoxin (CTX) injury repair (41). This method has been previously found to better resolve the satellite cell contribution to myogenesis (42) (Figure 2A). At sequential time points after a second injury, animals were sacrificed, and TA muscle histology was analyzed. Histologically, hypercapnic mice demonstrated a more disorganized early repair compared with wild-type littermates, with poorer demarcation of myofiber integrity and higher mononuclear cell infiltration at 4 days after injury in comparison with the normocapnia counterparts (Figure 2B). At 12 days after injury, both normo- and hypercapnic animals demonstrated recovery of myofiber integrity; however, myofiber cross-sectional area in hypercapnic mouse TA muscles was significantly reduced in comparison with the normocapnic littermates (Figure 2, C and D).

### Chronic hypercapnia leads to reduced satellite cell activation.

**Figure 3:**
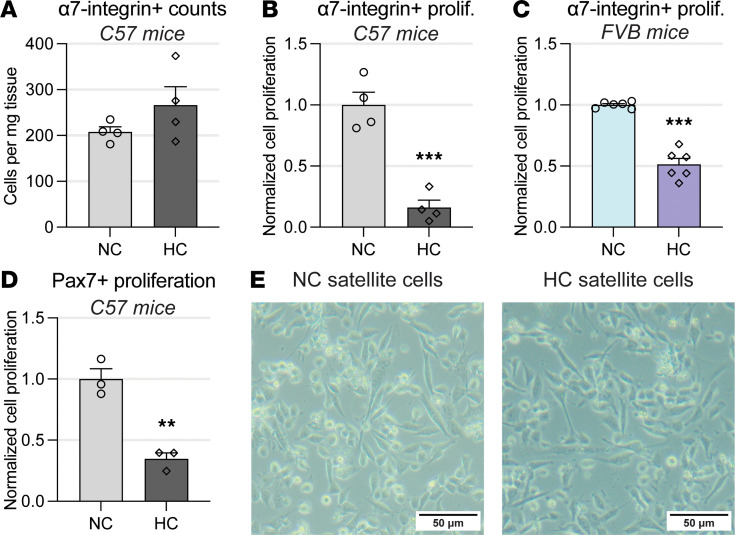
Chronic hypercapnia leads to reduced satellite cell activation. (**A**) Total numbers of α7-integrin–positive satellite cells isolated from skeletal muscles from NC and HC mice are similar (*n* = 4). (**B**) Column-isolated (MACS) α7-integrin–positive satellite cells from C57 HC mice have significantly reduced proliferation as measured by EdU assay in comparison with NC counterparts (*n* = 4). (**C**) FVB mice similarly exposed to hypercapnic conditions also display reduced α7-integrin satellite cell proliferation in reference to NC counterparts, indicating the process is not unique to the C57 strain (*n* = 6). (**D**) FACS-isolated Pax7-GFP–positive muscle satellite cells also demonstrate significant reduction in proliferation rate in reference to NC counterparts (*n* = 3). (**E**) α7-integrin–positive satellite cells from both NC and HC mice were plated and demonstrate unremarkable morphology and no evidence of cellular toxicity induced by hypercapnia. All statistical comparisons were performed using Student’s *t* test; **P < 0.01 and ***P < 0.001.

To investigate if the myogenesis impairment induced by hypercapnia was associated with an alteration in satellite cells’ activation, we evaluated freshly isolated satellite cells. Using epitope-specific isolated satellite cells (43), we found that the numbers of α7-integrin–positive, freshly isolated cells from uninjured hypercapnic muscles did not significantly differ from normocapnic counterparts (Figure 3A). Those data suggest that before injury, the number of satellite cells present in normo- and hypercapnia mice is similar. We then conducted experiments to evaluate the initial phase of myogenesis, which is the activation of satellite cells and their symmetrical replication (14, 44). To quantify the replication status of satellite cells isolated from normo- versus hypercapnic mice, we conducted a 5-ethynyl-20-deoxyuridine (EdU) incorporation assay (21). This method, which is based on EdU incorporation to the DNA during the S phase of the cell cycle, has been previously calibrated to resolve satellite cells’ replicative status at 40 hours after their isolation (45). We observed that satellite cells isolated from hypercapnic mice demonstrated a significantly lower replication rate in comparison with normocapnic littermates (Figure 3B). As the animal’s genetic background influences multiple properties of satellite cells (46), we sought to eliminate a strain-introduced bias. To do that, we repeated the same experiments, this time using FVB mice, which also demonstrated a significant reduction of satellite cells’ replication following hypercapnia (Figure 3C). The isolation of satellite cells by selecting surface expression of α7-integrin is a validated method (47–49). However, we posited that this isolation method could introduce an unknown bias, potentially confounding the observed outcomes. We then conducted an alternative method of cellular isolation via fluorescence-activated cell sorting (FACS) using the canonical satellite cell marker Pax7 (42). To do that, we used an animal with conditional green fluorescence protein (GFP) reporter in satellite cells. This animal was generated by crossing a mouse with an internal ribosome entry site–CreER^T2^ fusion protein inserted downstream of the stop codon of the *Pax7* gene with a mouse containing a *loxP*-flanked STOP cassette preventing transcription of a CAG promoter–driven enhanced GFP (EGFP) variant (ZsGreen1). The result of that crossing leads to green fluorescence after Cre recombination, which occurs in Pax7-expressing cells but is not conjugated to the Pax7 protein itself (50). This model verified that hypercapnia caused a reduction of satellite cell replication in comparison with normocapnia counterparts (Figure 3D). To rule out the possibility that an abnormality of the satellite cell integrity could impair their measured replication rate, we plated cells in culture to observe their morphology and did not notice differences between normo- and hypercapnic experimental conditions (Figure 3E). Taken together, the data suggest that a suboptimal myogenic response in hypercapnic animals is associated with reduced satellite cell replication rate but not preinjury reduced numbers or conspicuously abnormal morphology.

### After-injury satellite cell contribution to myogenesis is reduced in hypercapnia.

**Figure 4:**
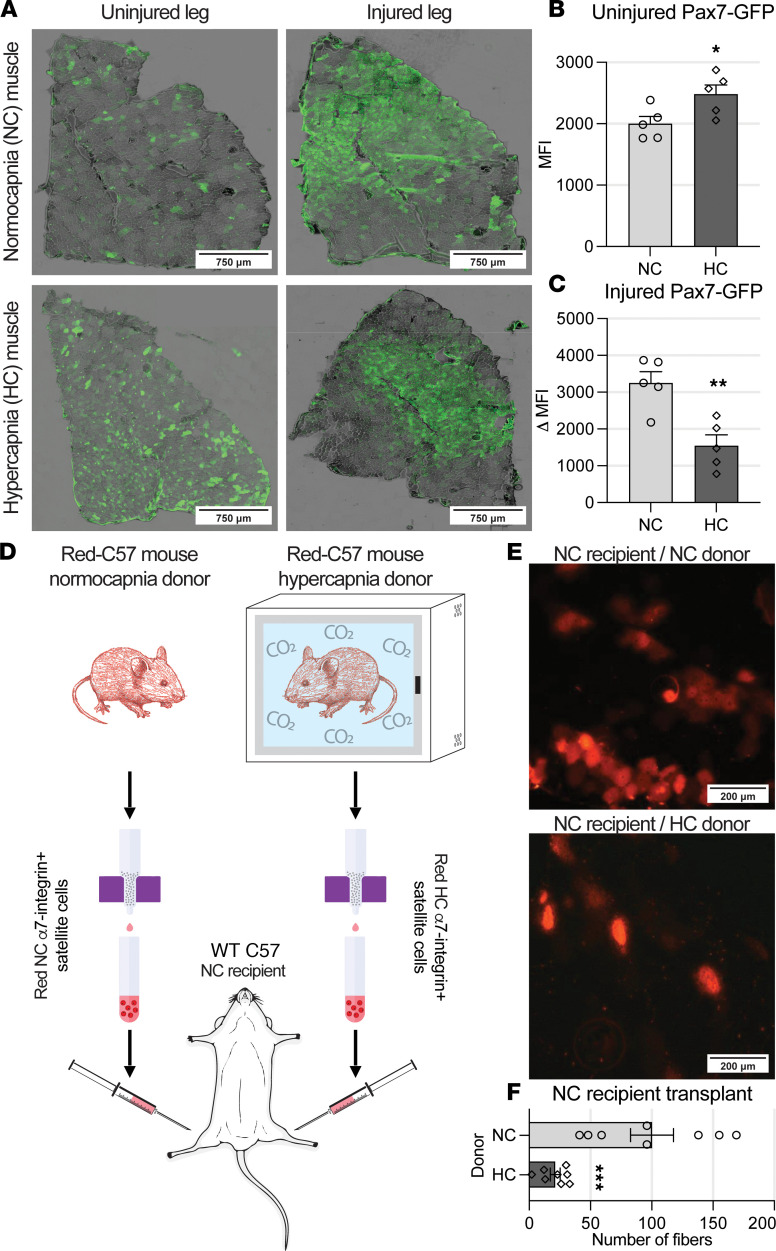
Postinjury satellite cells’ contribution to myogenesis is reduced in hypercapnia. (**A**) Pax7-GFP reporter mice induced with tamoxifen were exposed to normo- and hypercapnia, and TA muscle was then injured (once) with CTX to observe intrinsic satellite cell participation in muscle repair by section’s MFI. (**B**) Preinjury MFI is elevated in HC versus NC muscles (*n* = 5). (**C**) Postinjury muscle sections showed a significantly lower change in MFI compared with NC animals, indicating less Pax7-GFP cell participation in muscle repair (*n* = 5). (**D**) Graphic illustrates experimental design of transplantation experiments. (**E** and **F**) Column-isolated α7-integrin–positive satellite cells from RFP-expressing animals showed that HC mice produce satellite cells with significantly reduced myogenic capacity in comparison with NC counterparts, as determined by counting the number of red myofibers per TA muscle section 2 weeks after transplant into a healthy NC recipient. The animals’ contralateral legs were transplanted with cells from an NC donor as a control (*n* = 8). All statistical comparisons were performed using Student’s *t* test; *P < 0.05, **P < 0.01, and ***P < 0.001. Graphics from **D** were constructed with clipart supplied by BioRender.com.

To appreciate the myogenic effects of satellite cells in vivo, we used animals with conditional expression of Pax7-GFP, which label endogenous satellite cells with green fluorescence only after tamoxifen induction. After muscle injury, satellite cells become activated and thus undergo replication, eventually causing the myofibers fused with satellite cells to express green fluorescence indefinitely (50). TA muscles from Pax7-GFP animals previously exposed to normo- or hypercapnia were unbiasedly scored for mean fluorescence intensity (MFI) with and without previous administration of CTX-induced injury. To rule out interindividual variability, the change in MFI of the injured TA muscle was compared with the MFI of the contralateral leg’s uninjured muscle. While the preinjury MFI was slightly but significantly elevated in hypercapnic uninjured muscles, postinjury fluorescence was more significantly reduced in muscles from hypercapnic animals (Figure 4, A–C). The after-injury change in MFI (Δ MFI) is used as a surrogate of Pax7-GFP cell contribution to muscle repair. In this model, a bigger increase (Δ MFI) is associated with a more efficient satellite cell contribution to the reparative process (50). The data suggest that while baseline, preinjury hypercapnia could cause an increase in satellite cell turnover, chronic CO_2_ exposure reduces the after-injury satellite cell contribution to muscle repair, relative to normocapnia. Because the satellite cell contribution to myogenesis is influenced by the cellular surroundings, including the cell niche (15), we sought to determine intrinsic satellite cell myogenic potential in hypercapnia. To do that, we conducted satellite cell transplantation experiments. For these experiments, donor satellite cells were isolated from animals constitutively expressing red fluorescence protein (RFP) under control of the β-actin promoter (33, 51). After exposing RFP-expressing mice to normo- or hypercapnia, animals were sacrificed, and 50,000 cells were freshly isolated and transplanted into recipient normocapnic mice. As satellite cells from female mice replicate faster than (52), but engraft similarly to (53), male counterparts, donors were always sex matched. To reduce interindividual variability, we injected normo- and hypercapnic cells into right and left TA muscles, respectively, of the same animal (33, 34, 54) (Figure 4D). Given that freshly isolated satellite cells contribute to muscle repair by fusing with preexisting myofibers or with other satellite cells (15, 49), the number of RFP-expressing myofibers after muscle repair is, in this model, a surrogate of transplanted satellite cell–driven myogenesis (54). Two weeks after transplantation, recipient mice were sacrificed, and RFP-positive myofibers were counted. We found that normocapnia mouse–derived cells contributed to significantly more myofibers in comparison with hypercapnic donor cells (Figure 4, E and F). Phase contrast images indicated that transplantation experiments did not prevent full repair of muscle integrity (Supplemental Figure 1; supplemental material available online with this article; https://doi.org/10.1172/jci.insight.182842DS1). Together, the data suggest that satellite cell contribution to myogenesis is intrinsically disrupted in the context of chronic hypercapnia and persists so when the cell is removed from the hypercapnic environment.

### The transcriptomic landscape of hypercapnic satellite cells suggests an alteration of multiple metabolic and autophagy pathways.

**Figure 5:**
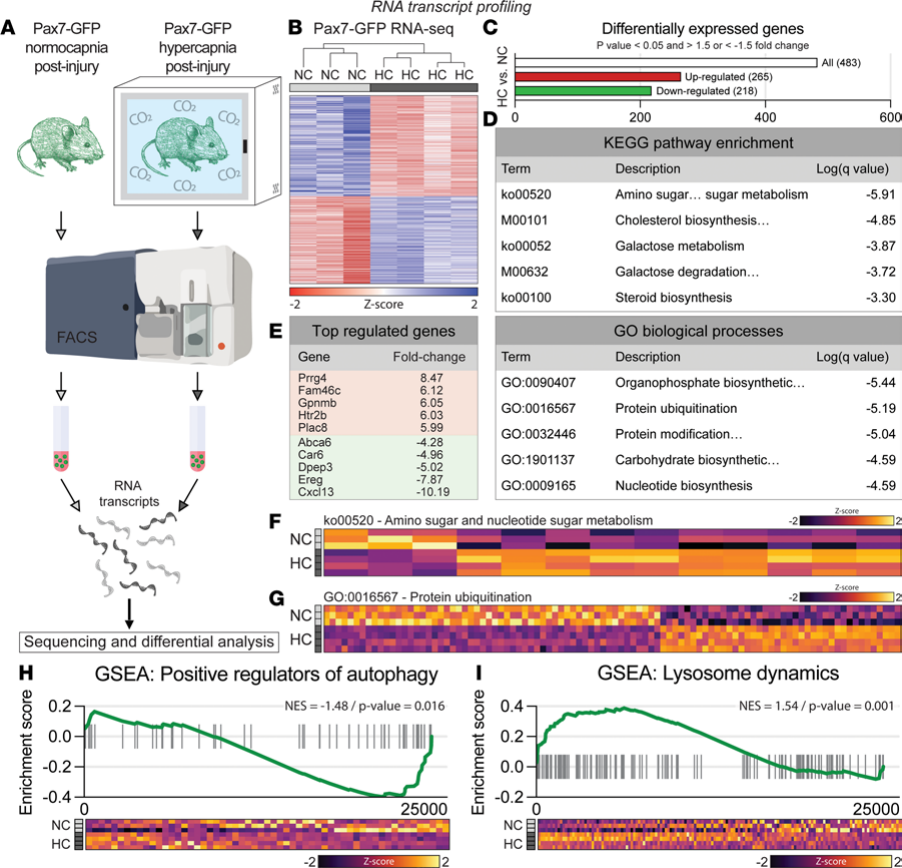
The transcriptomic landscape of hypercapnic satellite cells suggests alteration of multiple metabolic and autophagy pathways. (**A**) Graphic depicts the experimental design of the bulk RNA-sequencing analysis. (**B**) Heatmap of gene expression and unbiased clustering of RNA-sequenced samples (*n* = 3 NC, 4 HC). (**C**) Number of differentially expressed genes with a *P* < 0.05 and a fold-change greater than 1.5 or less than –1.5 shows 483 genes that are differentially expressed in hypercapnia versus normocapnia. (**D**) KEGG and GO pathways related to carbohydrate, amino acid and lipid metabolism, and protein ubiquitination. (**E**) List of top up- and downregulated genes. (**F** and **G**) Heatmaps show further interrogation of the number of entities within the top terms and their relative regulation in NC and HC mice. (**H** and **I**) GSEA of transcripts described as regulators of autophagy and lysosome dynamics. Graphics from **A** were constructed with clipart supplied by BioRender.com.

Because transplantation experiments suggested that satellite cells carried the defective myogenic potential into a normocapnic recipient, we reasoned that their transcriptional landscape would inform on mechanisms regulating their malfunction. Thus, both normo- and hypercapnic Pax7-GFP animals underwent muscle injury. After that, postactivation satellite cells were freshly isolated and sorted using FACS, followed by bulk RNA-sequencing analysis (Figure 5A and Supplemental Table 2). That analysis revealed unique transcriptomic profiles that clustered in discrete normo- versus hypercapnia groups involving 483 differentially expressed transcripts. In all, 265 transcripts were upregulated and 218 were downregulated in hypercapnia in comparison with normocapnia (Figure 5, B and C). Moreover, Kyoto Encyclopedia of Genes and Genomes (KEGG) and Gene Ontology (GO) enrichment analyses indicate strong signatures related to amino acid, sugar, and lipid metabolism (Figure 5, D and E, and Supplemental Table 3). A signature associated with protein ubiquitin modifications, which characterizes accelerated protein turnover, was highly dysregulated in hypercapnic cells (Figure 5, F and G). Given that we hypothesized that hypercapnia disrupts autophagy in satellite cells, we specifically investigated genes known to be regulated during autophagy induction. Using gene set enrichment analysis (GSEA), we queried pathways including positive regulators of autophagy and lysosome dynamics (55). While we found that both lysosomal dynamics and positive regulators of autophagy were significantly enriched in hypercapnia, positive regulators of autophagy were negatively enriched (Figure 5, H and I). This indicates that genes typically induced during autophagy activation were negatively regulated in hypercapnic satellite cells. These data suggest that after satellite cells become activated in the hypercapnic environment, transcriptomic changes are evoked in pathways related to metabolic substrate handling and ubiquitin modifications and negatively associated with autophagy function.

### Single-cell sequencing analysis of satellite cells, autophagy, and senescence.

**Figure 6:**
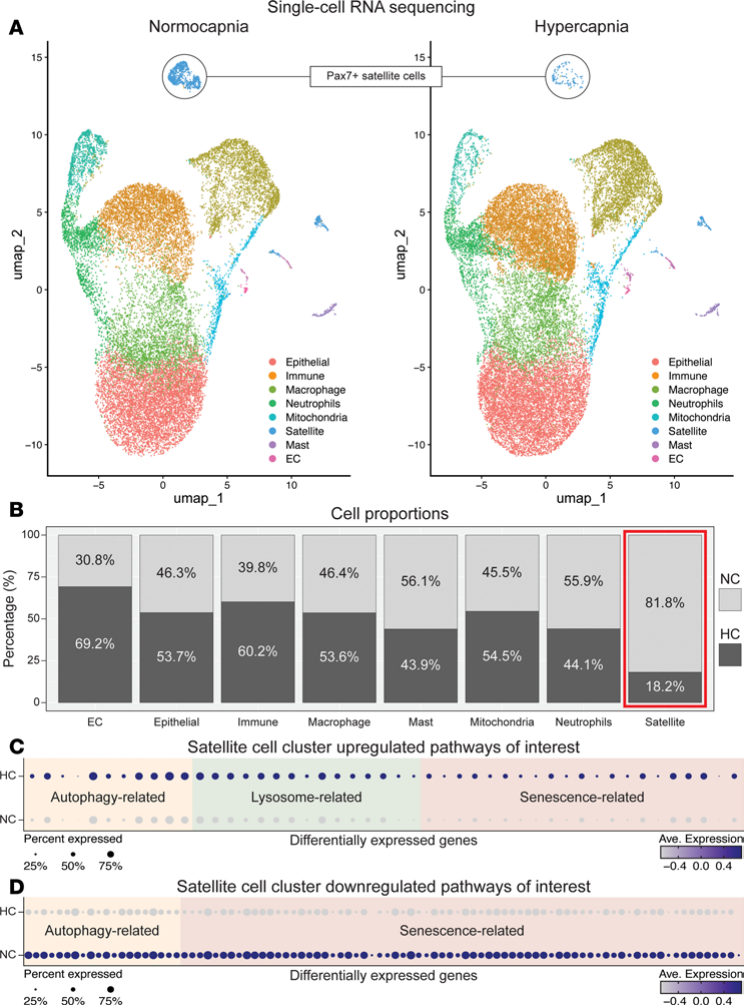
Single-cell sequencing analysis of satellite cells, autophagy, and senescence. Postinjury cells were sorted using FACS and processed for single-cell sequencing. (**A**) Uniform manifold approximation and projection (umap) of normo- and hypercapnia cell clusters identified. Note that hypercapnia caused a robust reduction in the number of satellite cells, with other cell types showing no such imbalance between normo- and hypercapnia conditions. (**B**) Cell proportions sequenced in normo- and hypercapnia. (**C** and **D**) In the specific satellite cell cluster, genes related to autophagy, lysosome, and senescence were identified. A total of 2 animals were sent for sequencing in each condition. EC, endothelial cells.

Bulk RNA sequencing aggregates multiple cell populations, which could hide potentially important signatures not expressed by most cells (56). Moreover, although the downregulation of autophagy in satellite cells has been associated with the development of senescence programs (22), the percentage of cells expressing senescence markers is frequently low (57). We then collected after-injury, activated satellite cells and conducted single-cell sequencing analysis. We identified a discrete cluster of cells expressing transcripts associated with satellite cells (Supplemental Figure 2 and Supplemental Table 4). Other identified clusters included cells expressing epithelial, endothelial, immune, and other inflammatory cell transcripts (Figure 6A). Significantly, hypercapnia caused a robust reduction in the number of after-injury satellite cells, with other cell types showing no such imbalance between normo- and hypercapnia conditions (Figure 6B). That reduction is likely contributed by the already-described reduced proliferation rate of hypercapnic satellite cells after their exit from quiescence. In the specific satellite cell cluster, we queried for genes related to autophagy, lysosome function, and senescence. We found multiple transcripts up- and downregulated in these analyses (Figure 6, C and D, and Supplemental Tables 5 and 6). These data indicate that following muscle injury in a hypercapnic condition, there is a reduction in satellite cell number and significant changes in multiple transcriptional programs that regulate autophagy and senescence in reference to normocapnia.

### Hypercapnia downregulates satellite cells’ autophagy in vivo.

**Figure 7:**
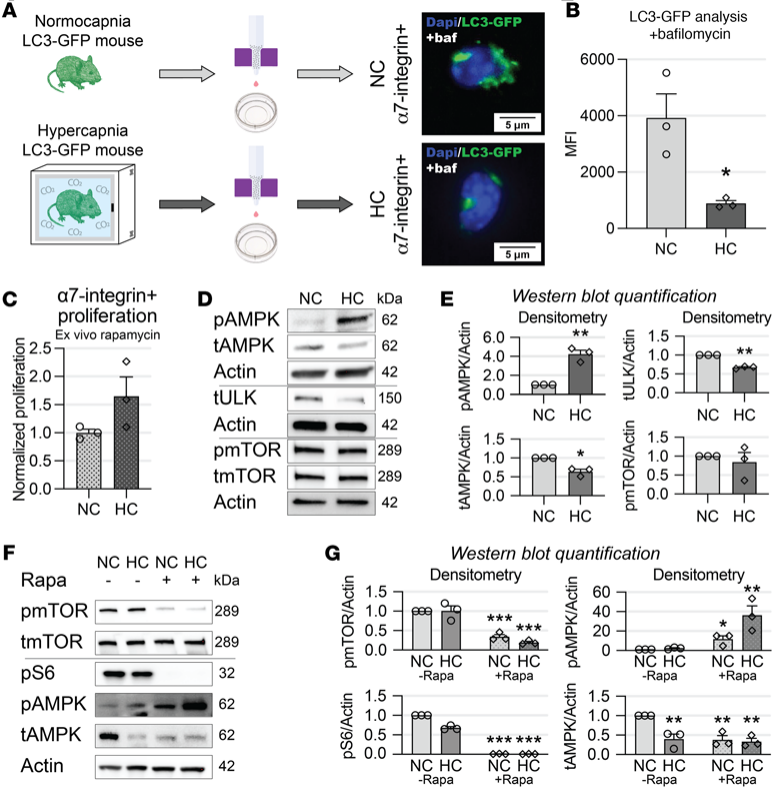
Hypercapnia downregulates satellite cells’ autophagy in vivo. (**A**) Diagram indicating the experimental design. (**B**) Freshly isolated α7-integrin–positive satellite cells obtained from LC3-GFP reporter HC mice show a significant reduction in puncta accumulation after bafilomycin treatment in reference to NC counterparts (*n* = 3). (**C**) Column-isolated α7-integrin–positive satellite cells from C57 HC mice regain higher proliferation capacity if treated with rapamycin as measured by EdU assay in comparison with HC counterparts not treated with that drug (*n* = 3). (**D**) Immunoblots of column-isolated α7-integrin–positive satellite cells from room air–breathing mice cultured for 4 days in NC and HC show that hypercapnia upregulates p-AMPK but causes a reduction of total AMPK and ULK1, without either translational or posttranslational effect on mTOR. Actin was used as a lane loading control; each lane corresponds to an individual mouse (*n* = 3). (**E**) Densitometric quantitation of Western blots in **D**. (**F**) Rapamycin administration causes a robust dephosphorylation of mTOR and ribosomal protein S6, which is associated with a further amplification of HC-induced upregulation of p-AMPK. (**G**) Densitometric quantitation of Western blots in **F**. Actin was used as a lane loading control; each lane corresponds to an individual mouse (*n* = 3). Statistical comparisons were performed using Student’s *t* test in **B** and **C**. Densitometric statistical comparisons in **E** and **G** were performed using a 1-sample *t* test (**E**) and 2-way ANOVA (**G**); *P < 0.05, **P < 0.01, and ***P < 0.001. Graphics from **A** were constructed with clipart supplied by BioRender.com.

Given the transcriptional regulation of autophagy-associated genes found in RNA-sequencing analyses, we then tested whether hypercapnia regulates satellite cells’ autophagy in vivo. To ascertain this, we used a mouse constitutively expressing an EGFP sequence fused with the LC3 family proteins, which retain fluorescence of autophagosomes after their formation and until they fuse with lysosomes (58). In this mouse model, autophagosome mass can be unbiasedly quantified in freshly isolated satellite cells by determining the MFI generated by GFP puncta (21, 59, 60) (Figure 7A). To evaluate the autophagosome turnover, freshly isolated and plated satellite cells obtained from normo- and hypercapnic mice were treated with bafilomycin, an autophagy flux inhibitor and vacuolar type H^+^-translocating ATPase inhibitor. This drug prevents lysosome degradation and thus leads to accumulating punctate GFP-LC3 exclusively when autophagy is active (22, 33, 61). These experiments revealed significantly reduced cytosolic MFI in satellite cells obtained from hypercapnic mice compared with normocapnic counterparts (Figure 7B), suggesting that hypercapnia downregulates autophagy flux in these cells. To test whether pharmacologic boosting of autophagy improves hypercapnic satellite cells’ replication, we treated cells obtained from normo- and hypercapnic mice with the allosteric mTOR pathway inhibitor rapamycin and found a not significantly different replication rate between the 2 conditions at 40 hours after isolation (Figure 7C). We then conducted experiments to gain insight into the signaling pathways associated with these processes in primary cells. Freshly isolated satellite cells obtained from room air–breathing mice were cultured ex vivo in normo- and hypercapnic conditions for 4 days. These conditions were generated by culturing cells in media buffered to maintain normal osmolarity, pH, and oxygen and either elevated or normal CO_2_, as previously published (6, 7). As early autophagy activation is in part regulated by the competitive effect between mTOR and AMPK on ULK1 (27), we probed immunoblots against these proteins’ products. We found that hypercapnia induced AMPK activation as measured by the surrogates of threonine 172 phosphorylation (AMPK^T172^, p-AMPK). However, the expression levels of both total AMPK and its targeted downstream effector Ulk1 were reduced. Interestingly, neither total nor phosphorylated mTOR at serine 2448 (mTOR^S2448^), which is a key regulator of mTOR activity (62), were influenced by hypercapnia (Figure 7, D and E). We then tested the regulation of these signaling pathways by rapamycin stimulation. Rapamycin caused mTOR^S2448^ and ribosomal S6 dephosphorylation (Figure 7, F and G), indicating that this cellular pathway can be pharmacologically targeted in satellite cells and primary myoblasts (22, 63, 64). Interestingly, while hypercapnia per se caused upregulation of AMPK^T172^ (Figure 7, D and E), that effect was amplified by concomitant exposure to rapamycin (Figure 7, F and G). This finding indicates that in satellite cells rapamycin inhibits mTOR and simultaneously stimulates AMPK, even in cells exposed to chronic hypercapnia.

### Rapamycin administration attenuates hypercapnia-induced impaired myogenesis in vivo.

**Figure 8:**
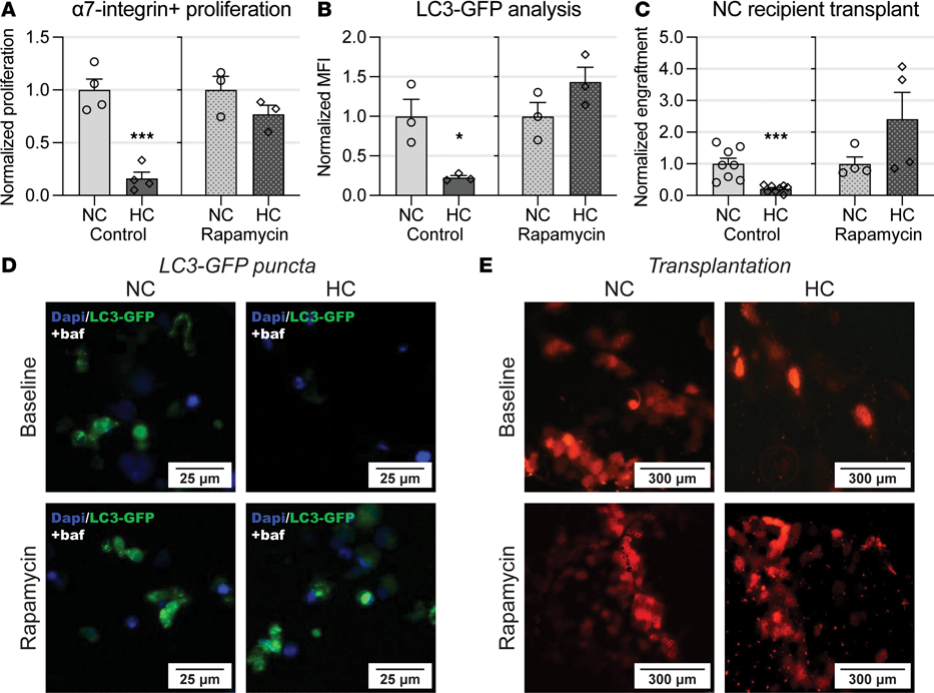
Rapamycin administration attenuates hypercapnia-induced impaired myogenesis in vivo. (**A**) Freshly isolated satellite cell proliferation is normalized by previous systemic rapamycin administration to HC mice (*n* = 3). (**B** and **D**) Hypercapnia-induced LC3-GFP puncta reduction is reversed by previous systemic rapamycin administration to HC mice (*n* = 3). (**C** and **E**) Transplantation experiments indicate that, after previous systemic rapamycin administration to HC mice, freshly isolated satellite cells engaged in posttransplantation, engraftment, and differentiation similarly to NC donated cells (*n* = 4). Note that control conditions of **A**–**C** (left bar graphs) correspond to experiments previously presented in the article and are displayed as a reference to rapamycin-administered mice. All statistical comparisons were performed using Student’s *t* test; *P < 0.05 and ***P < 0.001.

We then evaluated whether pharmacologic inhibition of the mTOR pathway could improve autophagy flux and myogenic potential in hypercapnic animals. To do that, we exposed mice to chronic hypercapnia and systemically treated them with rapamycin. In vivo administration of rapamycin prevented the inhibitory effect of hypercapnia on satellite cells’ replication rate (Figure 8A). We then evaluated autophagy flux, as measured by MFI in bafilomycin-treated satellite cells from LC3-GFP mice. We found that the reduced flux induced by hypercapnia was prevented in animals previously treated with rapamycin (Figure 8, B and D). Finally, we transplanted freshly isolated satellite cells obtained from normo- and hypercapnic RFP mice previously treated with rapamycin. Cells were engrafted into TA muscles of normocapnic recipient mice. The relatively reduced engraftment of cells obtained from hypercapnic compared with normocapnic animals was prevented by pretreating donor mice with rapamycin (Figure 8, C and E). These data indicate that the detrimental myogenic effects of chronic hypercapnia on skeletal myogenesis can be attenuated via systemic administration of rapamycin, which leads to an improved replication, autophagy flux, and myogenic potential of satellite cells.

### Hypercapnia downregulates autophagy, and autophagy knockdown replicates hypercapnia.

**Figure 9:**
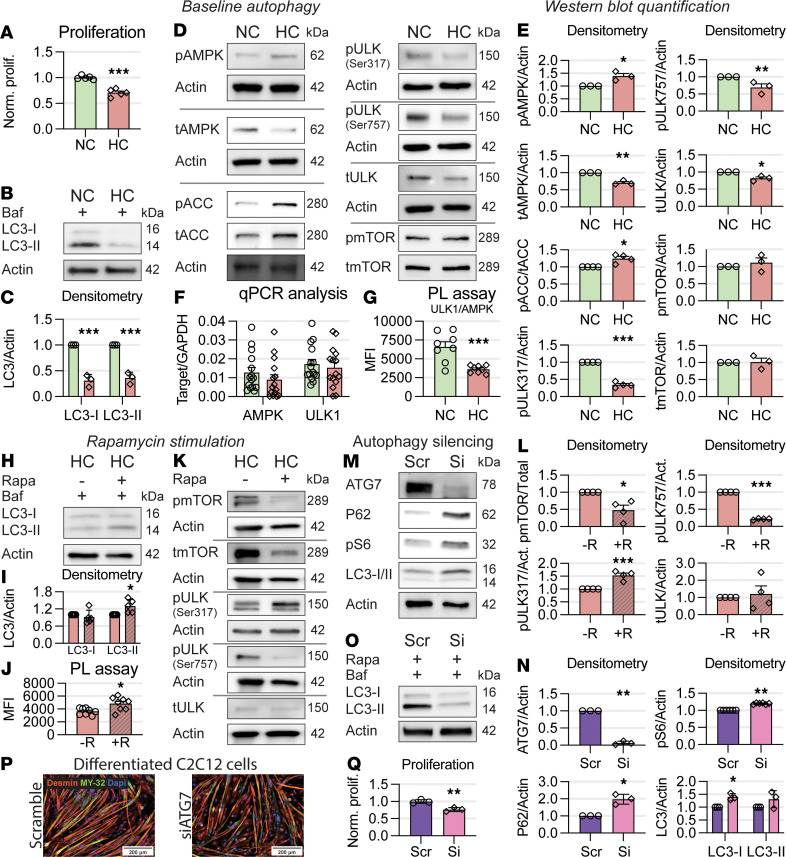
Hypercapnia downregulates autophagy, and autophagy knockdown replicates hypercapnia. (**A**) C2C12 myoblast proliferation is reduced in hypercapnia (*n* = 5). (**B**) Cells in hypercapnia express fewer LC3 isoforms (*n* = 3). (**C**) Densitometric quantitation of Western blots in **B**. (**D**) Autophagy pathway constituents are downregulated in hypercapnia; however, mTOR and p-mTOR (Ser2448) remain unchanged. Despite activation surrogates of AMPK (p-AMPK and p-ACC) increasing in hypercapnia, the canonical AMPK-targeted ULK1^S317^ is not upregulated (*n* = 3). (**E**) Densitometric quantitation of Western blots in **D**. (**F**) qPCRs of AMPK and ULK1 demonstrate lack of transcriptional downregulation (*n* = 16). (**G**) PLA shows that hypercapnia exposure is associated with reduced AMPK/ULK1-driven puncta (*n* = 8). (**H**) When treated with mTOR inhibitor rapamycin and bafilomycin, LC3-II increases in hypercapnia, suggesting retained autophagy flux capacity (*n* = 5). (**I**) Densitometric quantitation of Western blots in **H**. (**J**) PLA shows that hypercapnia-treated cells significantly improve AMPK/ULK1-driven puncta when treated with rapamycin (*n* = 8). (**K**) mTOR and downstream autophagy-inhibitory ULK1^S757^ are dephosphorylated after rapamycin treatment (*n* = 5), while AMPK-targeted and autophagy-stimulatory ULK1^S317^ are upregulated (*n* = 4). (**L**) Densitometric quantitation of Western blots in panel **K**. (**M**) siRNA Atg7 transfection in C2C12 cells causes a reduction in Atg7 protein products and upregulation of autophagy arrest markers. Scramble (scr) is control, nonspecific siRNA (*n* = 3). (**N**) Densitometric quantitation of Western blots in panel **M**. (**O**) C2C12 cells transfected with Atg7 siRNA are unable to build normal levels of LC3-II with rapamycin (*n* = 4). (**P**) C2C12 cells transfected with Atg7 siRNA demonstrated a normal differentiation pattern (*n* = 3). (**Q**) Atg7 silencing significantly reduces C2C12 cell proliferation similarly to hypercapnia (*n* = 3). Statistical comparisons were performed using Student’s *t* test in panels **A**, **F**, **G**, **J**, and **Q**. Densitometric statistical comparisons in **C**, **E**, **I**, **L**, and **N** were performed using a 1-sample *t* test; *P < 0.05, **P < 0.01, and ***P < 0.001.

Previously shown data suggest that hypercapnia downregulates myoblast proliferation, myogenesis, and autophagy. We then reasoned that if these processes were mechanistically associated, then autophagy knockdown (in normocapnic cells) should phenocopy key aspects of hypercapnia exposure. To conduct the following experiments, we used C2C12 myoblasts, which are nontransformed immortalized cells displaying features reminiscent of primary myoblasts, including their ability to replicate. These cells also differentiate into myotubes if cultured in serum-poor media (6, 7, 65). First, to corroborate the observed effects of hypercapnia on autophagy, C2C12 myoblasts were exposed to normo- and hypercapnia media as previously published (6, 7). CO_2_ exposure time course identified the time point when cells demonstrated a reduced replication rate evaluated with the EdU incorporation assay. We found that myoblasts exposed to hypercapnia for a minimum of 5 days demonstrated a significant reduction in cellular proliferation (Figure 9A and Supplemental Figure 3). As autophagy flux enables satellite cell activation (20, 21), we evaluated autophagosome turnover in normo- and hypercapnic cells. The ubiquitin-like protein Atg8 (LC3) can be conjugated to phosphatidylethanolamine, a process known as lipidation, that causes the transition from LC3-I to LC3-II. That transition is a proxy of autophagy flux when used along with bafilomycin, an autophagy flux inhibitor, which prevents lysosome degradation and thus causes LC3 lipidation to accumulate exclusively when autophagy is active (22, 61). Both LC3-I and -II were downregulated by hypercapnia exposure (Figure 9, B and C, and Supplemental Figure 4), suggesting a reduction in mass and lipidation of LC3 during hypercapnia. We then immunoblotted myoblast samples previously exposed to normo- and hypercapnia with antibodies against the mTOR and AMPK pathways. Consistent with primary myoblasts (Figure 7, D–G), we found that while hypercapnia exerted no effect on total or p-mTOR (mTOR^S1778^), it induced AMPK activation as measured by AMPK^T172^ (p-AMPK) and the AMPK downstream target acetyl-CoA-carboxylase (p-ACC). Similar to primary myoblasts, the expression levels of both total AMPK and its targeted downstream effector ULK1 were significantly reduced (Figure 9, D and E). Moreover, the phosphorylation level of ULK1 at serine-317 (ULK1^S317^), which is an AMPK-targeted site that induces autophagy (27), was reduced in hypercapnia. This last finding suggested that despite the increase in p-AMPK, the reduction of total AMPK was associated with a net-negative effect on ULK1^S317^ phosphorylation and with a reduced LC3 lipidation (Figure 9, B and C).

To gain insight into the mechanisms leading to reduced abundance of total AMPK and ULK1 in hypercapnia, we conducted quantitative PCR (qPCR) experiments using specific primers. Expression of AMPK and ULK1 mRNA products was not significantly reduced in hypercapnia, suggesting that the decreased protein abundances were caused by an accelerated degradation or a reduced synthesis but not transcriptional silencing (Figure 9F). Ubiquitin marks in cell lysates obtained from myoblasts treated with proteasomal inhibitor MG-132 were not significantly different between normo- and hypercapnic conditions (Supplemental Figure 5). Because AMPKa1 is the dominant isoform present in satellite cells and early differentiating myoblasts (66, 67), we conducted AMPKa1 immunoprecipitation from myoblasts treated with MG-132, with and without hypercapnia. These immunoblots were then probed with anti-ubiquitin antibodies. These data show that AMPKa1 ubiquitination did not increase by previous exposure to hypercapnia, suggesting that the reduction of AMPK abundance was not due to accelerated proteasomal degradation (Supplemental Figure 5). When AMPK phosphorylates and activates ULK1, these 2 proteins form a complex that enables autophagy initiation (27). Thus, we reasoned that the hypercapnia effect on autophagy would be associated with a reduction of that AMPK-ULK1 association. To interrogate this protein-protein interaction, we conducted proximity ligation assays (PLAs) using antibodies against ULK1 and AMPK. We indeed found that hypercapnia significantly reduced the puncta generated by AMPK’s proximity to ULK1, suggesting a diminished complexing between both proteins in that setting (Figure 9G and Supplemental Figure 6).

As we found that hypercapnia reduced myoblasts’ LC3 lipidation, we tested if treatment with rapamycin would reverse that. Indeed, rapamycin administration led to increased LC3 lipidation (Figure 9, H and I), which was associated with higher levels of AMPK/ULK1 complex formation in PLAs (Figure 9J) and mTOR deactivation as reflected by lower levels of p-mTOR and p-ULK1^Ser757^ (Figure 9K). Moreover, hypercapnic cells treated with rapamycin demonstrated increased levels of AMPK-targeted p-ULK1^Ser317^ (Figure 9K). Previous data indicate that AMPK and protein kinase B (Akt) antagonistically regulate skeletal muscle mass (6, 68, 69). Because Akt can inhibit autophagy (70) and regulate satellite cell stemness (71), we determined whether the Akt pathway was regulated in myoblasts exposed to elevated CO_2_. We found neither total nor p-Akt levels to be regulated by hypercapnia (Supplemental Figure 7). Also, in inflammatory cells, hypercapnia induced the expression of Bcl-2 and Bcl-xL, antiapoptotic factors that negatively regulate autophagy by blocking Beclin 1, an essential component of the autophagy initiation complex (24). To test if hypercapnia leads to similar regulation in myoblasts, we immunoblotted normo- and hypercapnic myoblast samples and probed them with antibodies against Bcl-2 and Bcl-xL. Our data show that none of the products were upregulated by myoblasts during hypercapnia, suggesting that a different mechanism is at play in the current setting (Supplemental Figure 8).

To test whether autophagy downregulation phenocopies key aspects of myoblast dysfunction evoked by hypercapnia, we knocked down autophagy and used EdU incorporation to score cell replication during normocapnia. To do that, we transfected C2C12 cells with control or specific siRNA against Atg7, which is a validated model of autophagy knockdown (72). These experiments revealed high efficiency of Atg7 downregulation and consistent p62 and p-S6 upregulation (Figure 9, M and N) (63), which were associated with a lack of rapamycin effect on LC3 lipidation in Atg7-knockdown cells (Figure 9O). Importantly, while autophagy knockdown did not influence myotube differentiation capacity (Figure 9P and Supplemental Figure 9), it led to a reduction in cellular proliferation (Figure 9Q). This is consistent with the phenotype observed in animals exposed to hypercapnia (Figures 3 and 4). In summary, these data indicate that hypercapnia inhibits autophagy and proliferation in C2C12 myoblasts and that autophagy silencing in C2C12 cells phenocopies key aspects induced by hypercapnia.

## Discussion

Here, we show that hypercapnia reduces satellite cells’ autophagy flux, proliferative rate, and myogenic potential. Administration of rapamycin is associated with improvement of these outcomes. Previous data indicate that hypercapnia accelerates the loss of muscle mass (6, 7). While hypercapnia and muscle wasting are independently associated with higher mortality in critically illness (1, 2, 5), human and experimental data suggest that impaired satellite cell function and myogenesis may contribute to muscle wasting in that setting (9–13). While autophagy flux is required to maintain satellite cell function and muscle mass (21, 22, 73), multiple models of critical illness have shown substantial dysregulation of autophagy in skeletal muscle (74–77). Previous evidence suggests that hypercapnia inhibits autophagy in inflammatory cells (24).

Myogenesis, which contributes to skeletal muscle integrity by enabling muscle repair following injurious events (42), depends in part on autophagy flux, which provides the bioenergetic needs for satellite cells’ activation and prevents their transition into senescence (20–23, 78–80). Previous studies have indicated that autophagy dysfunction causes abnormal myogenesis in aging (22) and in muscle dystrophy (20), yet to our knowledge this is the first study addressing autophagy regulation of myogenesis in the context of hypercapnia.

During an injurious event, satellite cells undergo an initial phase of activation followed by symmetrical replication giving rise to similar daughter cells, which expand the myogenic pool (44). After that, asymmetric division in an apical-basal orientation gives rise to 2 daughter cells, only one of which activates the transcription factor Myf5 and commits to myogenic differentiation (15). Our data suggest that it is the early activation with delayed symmetrical cell division which is primarily disrupted in the hypercapnic muscle response to injury. A previous report using C2C12 cells exposed to hypercapnia has suggested a reduced fusion index of these cells (18). Future studies with specific instruments to address asymmetrical cell division, such as the Myf5-YFP reporter mouse (81), will be needed to address the possible effect of elevated CO_2_ on the early phase of satellite cells’ differentiation.

To better analyze the contribution of satellite cells to the muscle reparative process in hypercapnia, we crossed an animal that conditionally expresses a GFP cassette controlled by a CAG promoter with an animal with a conditional Cre recombinase expression located at the Pax7 locus. The resulting mouse develops green fluorescence in Pax7-expressing satellite cells and in myofibers in which repair was contributed by these cells, even after Pax7 is no longer expressed. Decreased fluorescence was found in hypercapnic after-injury myofibers, which indicates that the myogenic potential of Pax7-expressing hypercapnic cells is reduced following an injurious event. Interestingly, preinjured muscles from hypercapnic animals showed a slightly yet significantly higher fluorescence (Figure 4B), possibly suggesting that hypercapnia is, per se, causing some level of recruitment and activation of satellite cells. Previous evidence suggests that skeletal muscles from chronically hypercapnic animals demonstrate hallmarks of recent myofiber regeneration (6). Future studies, including common pathways regulated by hypercapnia across different tissues and species (82–86), will be needed to specifically address satellite cells’ turnover in uninjured hypercapnic muscles.

Myogenesis is a complex process that involves the satellite cell and its tissue surrounding, including the cell’s niche (15). To investigate the specific contribution of satellite cells to hypercapnia-induced impaired myogenesis, we conducted transplantation experiments using constitutively fluorescent satellite cells obtained from normo- and hypercapnic mice. These cells were engrafted into a normocapnic animal, and their myogenic potential was quantified with lineage tracing. These experiments showed that hypercapnic satellite cells have significantly lower myogenic potential relative to normocapnic cells (Figure 4, E and F). These findings suggest that a satellite cell abnormality contributes to dysfunctional myogenesis and that these alterations are not rapidly reversible by engraftment into a normocapnic recipient. The identification of hypercapnia-induced satellite cell dysfunction could be therapeutically relevant given the potential of satellite cells’ isolation, expansion, and transplantation into a diseased muscle to improve clinical outcomes (87). It should be noted that transplanted satellite cells are obtained from a cellular niche exposed to other cell types, including inflammatory and immune cells (86, 88), that could also influence their function (15).

To further investigate the mechanisms of dysfunctional myogenesis in response to hypercapnia, we conducted bulk transcriptomic analysis of freshly isolated satellite cells from normo- and hypercapnic animals. We found that most of the dysregulated biological processes in hypercapnia were related to metabolic, protein ubiquitin, and autophagy pathways. As bulk sequencing analysis often hides subtle changes that could be impactful on cellular outcomes, and most importantly, does not discriminate transcripts contributed by multiple cell types (89), we conducted single-cell sequencing analysis of after-injury, freshly isolated and fixed satellite cells. This analysis revealed fewer after-injury satellite cells in hyper- versus normocapnic mice, likely due to a delayed activation and reduced symmetrical cell division shown by the EdU incorporation assay. This analysis also indicates that both autophagy and senescence-associated genes are relatively dysregulated in satellite cells obtained from hypercapnia mice. Consistently, previous research found that aging is associated with a p16^INK4a^-regulated transition from quiescence to senescence and with a reduction of satellite cells’ number and autophagy flux (22, 78). As abnormal autophagy is a central hallmark of aging (90), the presence of senescence features in hypercapnic satellite cells suggests that elevated CO_2_ may cause an accelerated aging in these cells.

The relationship between cellular metabolism and autophagy is complex and not fully understood. While the increase in glucose-derived acetyl-CoA inhibits autophagy (91), autophagy is critical to maintain the substrate availability needed to support the early satellite cell’s function (21). Multiple substrate-responsive signaling molecules regulate autophagy, including AMPK, mTOR, and protein kinase B (Akt) (21, 92, 93).

The effect of autophagy impairment in CO_2_-induced dysfunctional myogenesis has never to our knowledge been tested in vivo. To do that, we used an animal expressing the EGFP fused with the LC3 family proteins, which retain constitutive fluorescence of autophagosomes after their formation until they fuse with lysosomes (58). In this model, autophagosome mass can be unbiasedly scored in freshly isolated satellite cells by determining the MFI generated by GFP puncta (21, 59, 60). Freshly isolated satellite cells from normo- and hypercapnic mice were isolated and treated with bafilomycin, verifying autophagy flux deceleration in elevated CO_2_. This phenomenon occurs despite animals’ reduced food intake, which is expected to accelerate autophagy (61, 94). This suggests a robust effect of CO_2_, which is able to overcome an antagonistic fasting-induced autophagy activation.

The role of AMPK in this model is especially intriguing. AMPK has been previously shown to support both satellite cell activation (67) and autophagy (27), and hypercapnia is a powerful inducer of AMPK activation, both in previous research (6, 7, 95, 96) and in the current experiments. Yet, according to the data presented here, hypercapnia inhibits autophagy. This inhibition is associated with downregulation of multiple constituents of the autophagy initiation machinery, including AMPK, ULK1, and LC3. Importantly, hypercapnia exerts no apparent effect on the canonical autophagy inhibitor mTOR. We reasoned that the lack of hypercapnia effect on mTOR offered an opportunity to recruit that pathway with rapamycin to offset the effects of reduced autophagy constituents, thereby increasing autophagy flux in satellite cells. Our results from hypercapnic cells and animals treated with rapamycin indeed show an attenuation of multiple surrogates of autophagic and myogenic dysfunctions.

Autophagy induction depends on 2 competing signals on ULK1: AMPK is stimulatory, and mTOR is inhibitory (27). ULK1^S317^, a phosphorylation site targeted by AMPK that causes autophagy activation (27), was found to decrease in hypercapnia. Thus, we reasoned that despite CO_2_-driven increased AMPK^T172^ phosphorylation, the simultaneous reduction of total AMPK abundance seemed to lead to net autophagy inhibition. Consistently, the autophagy-inducing complex formation of AMPK and ULK1 was found to be reduced by hypercapnia.

**Figure 10:**
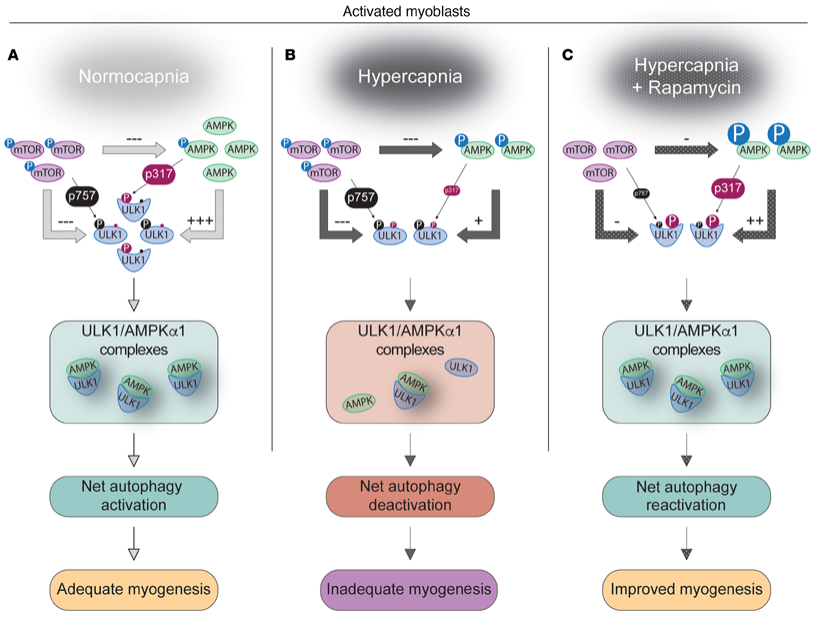
Proposed model accounting for the effects of chronically elevated CO_2_ on satellite cells’ activation and skeletal myogenesis and the effect of rapamycin administration. (**A**) During normocapnia (NC) and after satellite cell activation (induced by injury), autophagy flux supports myogenesis because of a shift in balance between the stimulatory effect of AMPK on ULK1 (indicated by ULK1^S317^ as a surrogate of this signaling) that overcomes the inhibitory effect of mTOR on ULK1 (indicated by ULK1^S757^ as a surrogate of this signaling). (**B**) In hypercapnia, both AMPK and ULK1 expression levels, AMPK/ULK1 complexing, and ULK1^S317^ are reduced. The inhibitory ULK1^S757^ remains unchanged. That rebalancing leads to the predominance of the inhibitory mTOR pathway (due to reduced AMPK effect), causing net autophagy deceleration and impaired myogenesis. (**C**) If HC animals are treated with rapamycin, the inhibitory ULK1^S757^ signaling is sharply downregulated, and the AMPK phosphorylation is robustly increased, leading to the elevation of the surrogate autophagy activation marker ULK1^S317^, and increase in AMPK/ULK1 complex formation, which indicates a resetting of the balance back to autophagy acceleration and improved myogenesis. Note that this is a proposed, not a factual model. See Discussion for further details.

Surprisingly, neither total mTOR nor p-mTOR^S2448^ was regulated by hypercapnia, suggesting that the effects of a weaker net AMPK signaling were unopposed by reciprocal mTOR compensation. In other words, given that the AMPK/ULK1 axis canonically activates autophagy while mTOR antagonizes it, we speculate that during satellite cells’ activation in the hypercapnic setting, reduced expression of AMPK and ULK1 along with preservation of mTOR’s expression turn the steady-state balance toward a net deactivation of autophagy flux (graphic of the proposed model in Figure 10). This postulation is also suggested by a reduction in LC3 and the subsequent lipidation to LC3-II, indicating a deceleration of autophagy flux induced by hypercapnia. This suggested model should, however, be interpreted as a postulation, not as factual, and thus warranting further research. Indeed, AMPK targets multiple phosphorylation sites on ULK1 (97), and thus, future experiments will be needed to determine the protein modifications induced by hypercapnia on ULK1. Moreover, studies involving loss of these modifications via site-specific mutagenesis will be needed to mechanistically resolve the abnormal autophagy regulation in hypercapnia.

Given that hypercapnia caused a reduced autophagy flux but did not regulate the antagonistic mTOR pathway, we tested whether the use of rapamycin to deactivate mTOR during hypercapnia would lead to net autophagy acceleration. Indeed, rapamycin treatment antagonized the inhibitory mTOR signaling along with further decrease in mTOR-targeted p-ULK1^S757^. This was associated with acceleration of LC3-II formation, AMPK/ULK1 complex formation, and increase in AMPK-targeted p-ULK1^S317^, suggesting that cells chronically exposed to hypercapnia still retain the ability to activate autophagy upon mTOR pathway inhibition.

The abnormal signaling pathways we observed in cultured C2C12 myoblasts were replicated in hypercapnic primary cells, and indeed rapamycin administration led to mTOR and ribosomal S6 protein dephosphorylation, acceleration of cell replication, autophagy flux, and satellite cells’ myogenic potential following transplantation and lineage tracing. Moreover, we found that rapamycin amplifies the CO_2_-induced AMPK activation, which may potentially account for the AMPK targeting of ULK1^S317^ associated with acceleration of autophagy flux and replication rate of satellite cells. A mechanism depending on mTOR’s direct targeting of AMPK has been recently described to play a critical role in the regulation of autophagy under nutritional stress (98). Future studies with site-specific mutagenesis could define if these or other residues are causally relevant in AMPK-mediated autophagy acceleration after rapamycin administration.

To determine if autophagy deceleration and hypercapnia exposure were mechanistically related, we reasoned that autophagy knockdown should phenocopy the CO_2_-induced processes. Myoblasts were then transfected with Atg7 siRNA, which caused a robust autophagy knockdown demonstrated by reduction of Atg7 expression and by upregulation of both p62 and ribosomal p-S6 (22, 73). Moreover, rapamycin administration to Atg7-knockdown cells was unable to increase LC3 lipidation, strongly suggesting an efficient loss of autophagy function. Importantly, Atg7-knockdown cells demonstrated a reduction in proliferation rate and retained differentiation capacity, which mimics the phenotype evoked by hypercapnia.

The mechanism articulating autophagy arrest with reduced satellite cell replication and myogenesis in hypercapnia remains to be elucidated. Previous research indicates that inhibition of autophagy suppresses ATP levels needed for satellite cells’ activation, which is partially rescued by exogenous pyruvate supply as an energy source (21). The interaction between mitochondrial integrity and autophagy flux has been demonstrated in other models (23, 79, 80) and could be relevant here as well. While our data suggest that hypercapnia-induced abnormal satellite cell function contributes to reduced myogenesis, future research will be needed to substantiate the proposed hypothesis in which rapamycin resets the balance toward higher AMPK and lower mTOR signaling operating on ULK1 (Figure 10). Moreover, while we show that rapamycin administration resulted in higher levels of AMPK-targeted p-ULK1^S317^, we used that phosphorylation site as a surrogate of activation; yet, we are not certain that ULK1^S317^ is indispensable, or exclusively required, for autophagy activation.

A question that remains unanswered is whether the effects of hypercapnia on myogenesis are dose dependent and can thus be replicated with lower levels of CO_2_ exposure, as previously shown in differentiated myotubes (6). The present study was designed to address the effects of hypercapnia on myogenesis after muscle injury and the relevance of autophagy in that setting. However, the observation that satellite cells’ fusion with uninjured myofibers appears to occur more significantly in hypercapnia relative with normocapnia might reflect an increased muscle turnover induced by chronic CO_2_ exposure. Although addressing this finding is beyond the scope of the present project, this could be investigated with future research. Although hypercapnia causes muscle wasting (6, 7), it is possible that other body mass constituents, such as adipose tissue and bone, are influenced in that setting. Importantly, observations suggest that while subcutaneous adipose tissue mass is not associated with worse outcomes in critical illness (1, 2), the effect of visceral and ectopic fat appears to be detrimental (99, 100). Thus, investigation of hypercapnia effects on adipose tissue turnover in critical illness will have to account for regulation exerted on unique depots.

Finally, while hypercapnia effects on myogenesis appear to involve AMPK-mediated regulation, this process could be influenced by phenomena taking place in other organs. For instance, evidence indicates that elevated CO_2_ causes AMPK-mediated downregulation of sodium transporters in alveolar cells, which decreases alveolar fluid clearance. It therefore could be possible that the alveolar fluid clearance, not investigated in our model, could introduce a bias to the present findings (101–103).

In summary, pharmacologic mTOR pathway inhibition attenuates hypercapnia-induced autophagy arrest in satellite cells and dysfunctional myogenesis, which could be relevant to improve clinical outcomes of patients with muscle wasting in CO_2_-retaining pulmonary diseases.

## Methods

Further information can be found in Supplemental Methods. Target-specific primers are available in Supplemental Table 1. Western blotting antibodies are available in Supplemental Table 7.

### Sex as a biological variable

Both male and female mice were used for all experiments in this study.

### Statistics

Data are expressed as the means ± SEM. When results were compared with a reference value, we used a 2-tailed 1-sample *t* test; when comparisons were performed between 2 groups, significance was evaluated by 2-tailed *t* test, and when more than 2 groups were compared, 2-way ANOVA was used followed by the Dunnett test using GraphPad Prism software. Small sample sizes (<5–10) are not informative in determining data normality. Where possible, normality was tested using the Shapiro-Wilk test. For experiments with sample sizes less than 5, normal data distribution for statistical tests used to determine significance was assumed. A *P* value lower than or equal to 0.05 was considered statistically significant.

### Study approval

All the procedures involving animals were approved by the Albany Medical College Institutional Animal Care and Use Committee (number 22-06002). Animals were handled according to the NIH *Guide for the Care and Use of Laboratory Animals* (National Academies Press, 2011), and all methods were performed in accordance with the relevant guidelines and regulations, as stated by the Journal and public agencies.

### Data availability

#### Bulk transcriptomics.

All the datasets including the raw files can be accessed via the accession number GSE226928.

#### Single-cell transcriptomics.

All the datasets including the raw files can be accessed via the accession number GSE266996**.**

#### Supporting data.

All supporting data values associated with the main manuscript and supplement material including values for all data points shown in graphs and values behind any reported means are available in the Supporting Data Values Excel file.

Prior publication: Part of this study has been previously presented in an abstract form at the 2023 American Thoracic Society Meeting on May 19–24, 2023, in Washington DC, USA.

## Author contributions

JB, LAD, ELJ, and DVS performed experiments; JB and RBR conducted bulk and single-cell RNA-sequencing analyses; and JB, HAS, and AJ designed the experiments and wrote the current manuscript.

## Supplementary Material

Supplemental data

Unedited blot and gel images

Supplemental tables 1-7

Supporting data values

## Version 1 11/26/2024

In-Press Preview

## Version 2 01/09/2025

Electronic publication
