# Supplementary material for: Rapamycin improves satellite cells’ autophagy and muscle regeneration during hypercapnia
Source: JCI Insight. 2025 Jan 9;10(1):e182842. doi: 10.1172/jci.insight.182842 (PMC11721297; doi:10.1172/jci.insight.182842)
Supplement: Supplemental data [file jciinsight-10-182842-s061.pdf]

Supplemental Data legends:

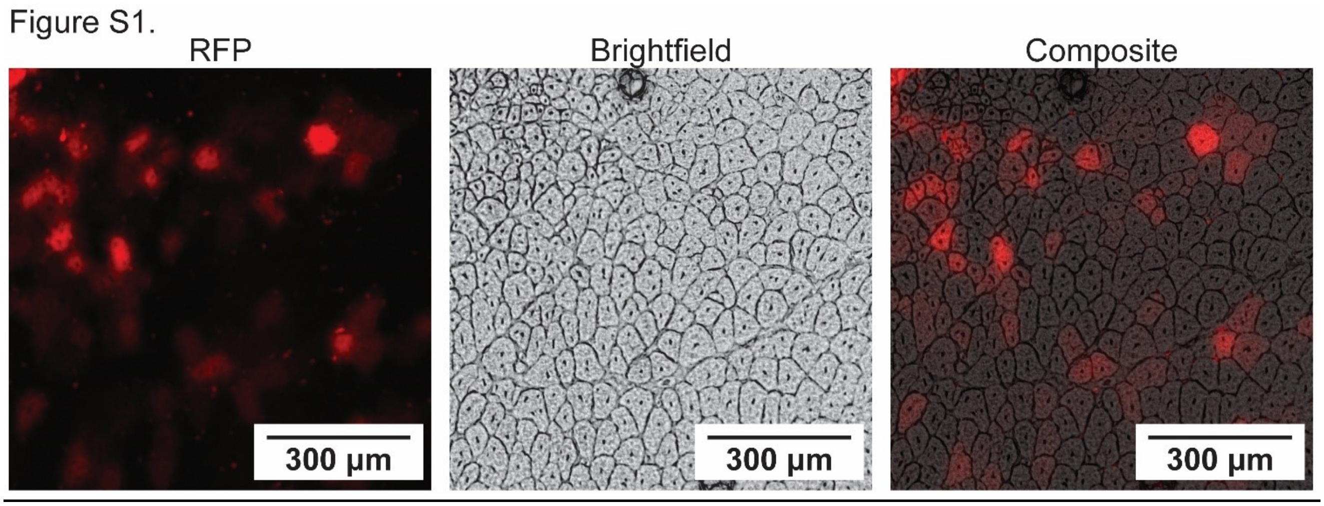

S1: **Muscle integrity post-transplant**. Contrast phase images demonstrating that transplantation experiments allow for full muscle regeneration after engraftment. Notice the central localization of the nuclei, which are hallmarks of the ongoing injury-repair cycle with recovery of sarcolemma integrity.

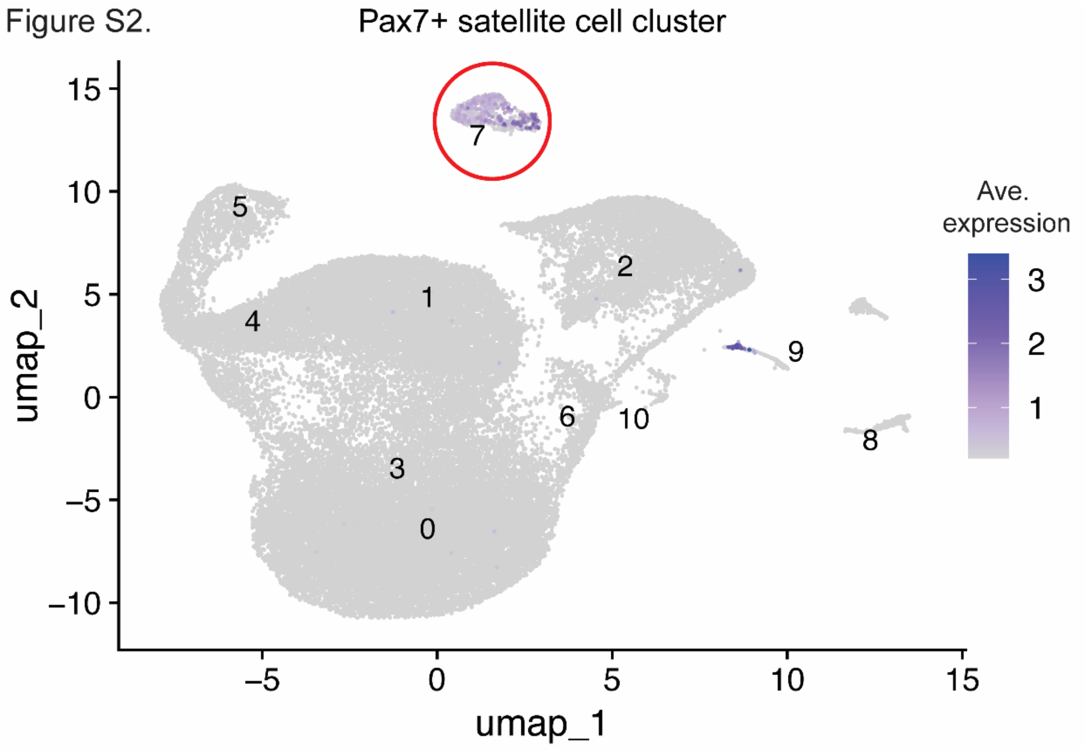

S2: Single cell sequencing aggregated umap plot. Average Pax7 transcript expression across all identified clusters. Cluster 7, circled in red, is the only cluster identified as Pax7+ satellite cells.

Figure S3.

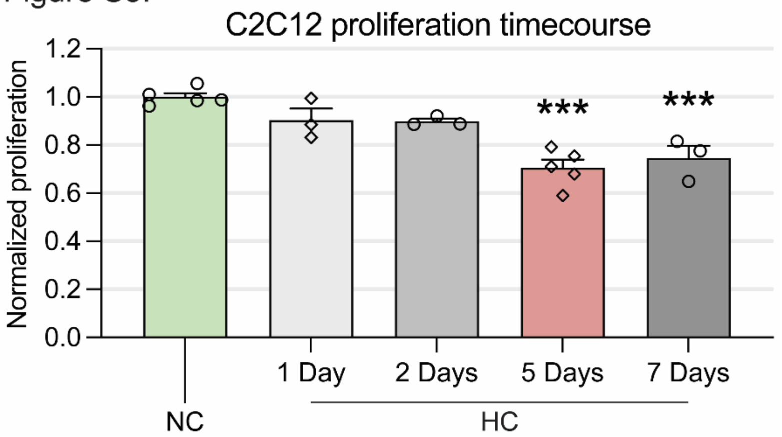

S3: **Chronic hypercapnia model in vitro**. Time-course of EdU incorporation assay of C2C12 cells exposed to hypercapnia. C2C12 myoblasts were maintained in NC or HC media and EdU incorporation was quantified at specific timepoints, indicating that at 5 days cells already demonstrate a significantly reduced replication rate in comparison with hypercapnia and normocapnia. N=5, *** $p<0.001$.

Figure S4.

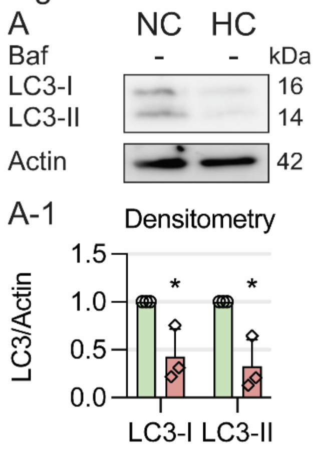

S4: C2C12 cells autophagy flux interrogation without bafilomycin. Myoblast untreated with autophagy flux inhibitor bafilomycin were exposed to hypercapnia and then lysed and processed for Westen blot using anti-LC3-specific antibodies. Actin was used as a lane loading control. N=3, $p<0.05$.

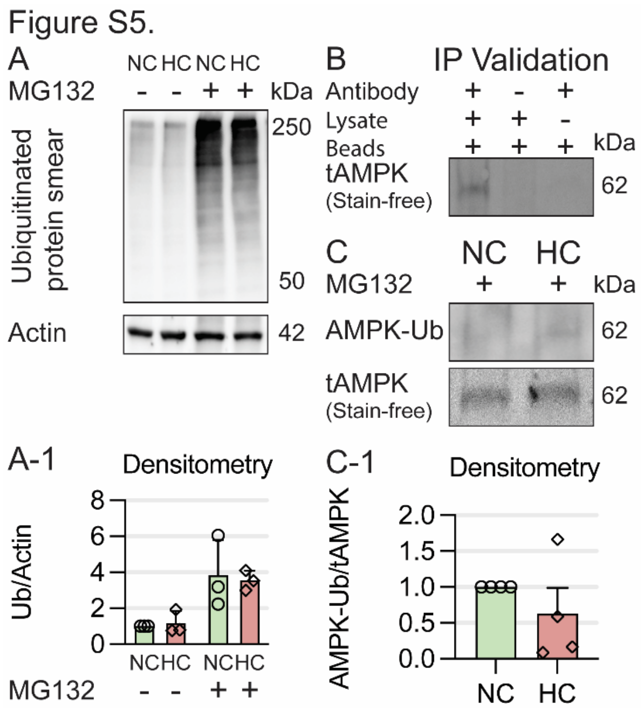

S5: Proteasome-mediated degradation in hypercapnic myoblasts. **A**: Normo and hypercapnic C2C12 myoblasts treated with proteasome inhibitor MG-132 were lysed and processed for Western blot using anti ubiquitin antibody. Note that while MG-132 causes acumination of ubiquitinated protein, there is no noticeable difference between normo and hypercapnic groups. N=3. **B**: Immunoprecipitation validation of AMPKα1 showing the enrichment of the band corresponding to the antibody/lysate/beads, which indicates the specific nature of the pulldown. **C**: C2C12 myoblasts exposed to normo and hypercapnia were processed for immunoprecipitation using AMPKα1 antibodies, and membranes were probed with anti-ubiquitin antibody. Data demonstrates that AMPKα1 does not significantly conjugate with ubiquitin in the presence of the proteasome inhibitor MG-132. N=4.

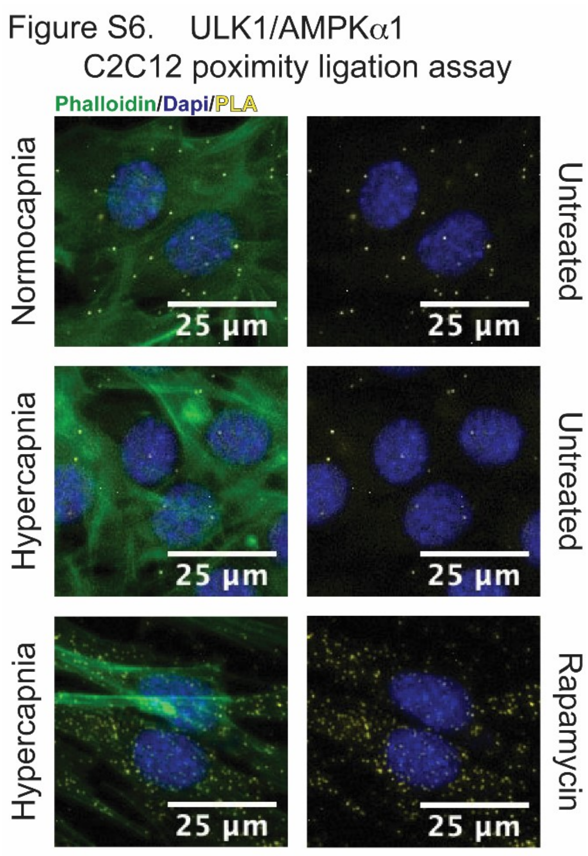

S6: Proximity ligation assays (PLA): C2C12 myoblasts were treated with normo and hypercapnia, and then hypercapnia with rapamycin; and then processed for PLA using primary AMPKa1 and ULK1 antibodies. Amplification probes conjugated to the secondary antibodies led to the fluorescent puncta formation. See further explanation in the methods section.

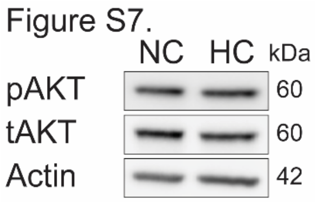

S7: **Effect of hypercapnia on Akt**. Hypercapnia does not regulate Akt total or phosphorylated forms. C2C12 cells in normo and hypercapnia were probed with total and phospho Akt (Ser473), which showed no regulation of any product induced by elevated CO_2. N=3

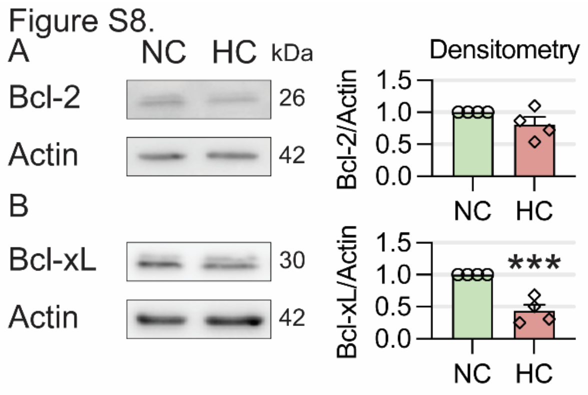

S8: Effect of hypercapnia on Bcl-2 and Bcl-xL: Myoblast were exposed to hypercapnia and then lysed and processed for Westen blot using anti-Bcl-2 and Bcl-xL-specific antibodies. Actin was used as a lane loading control. N=3, p<0.001

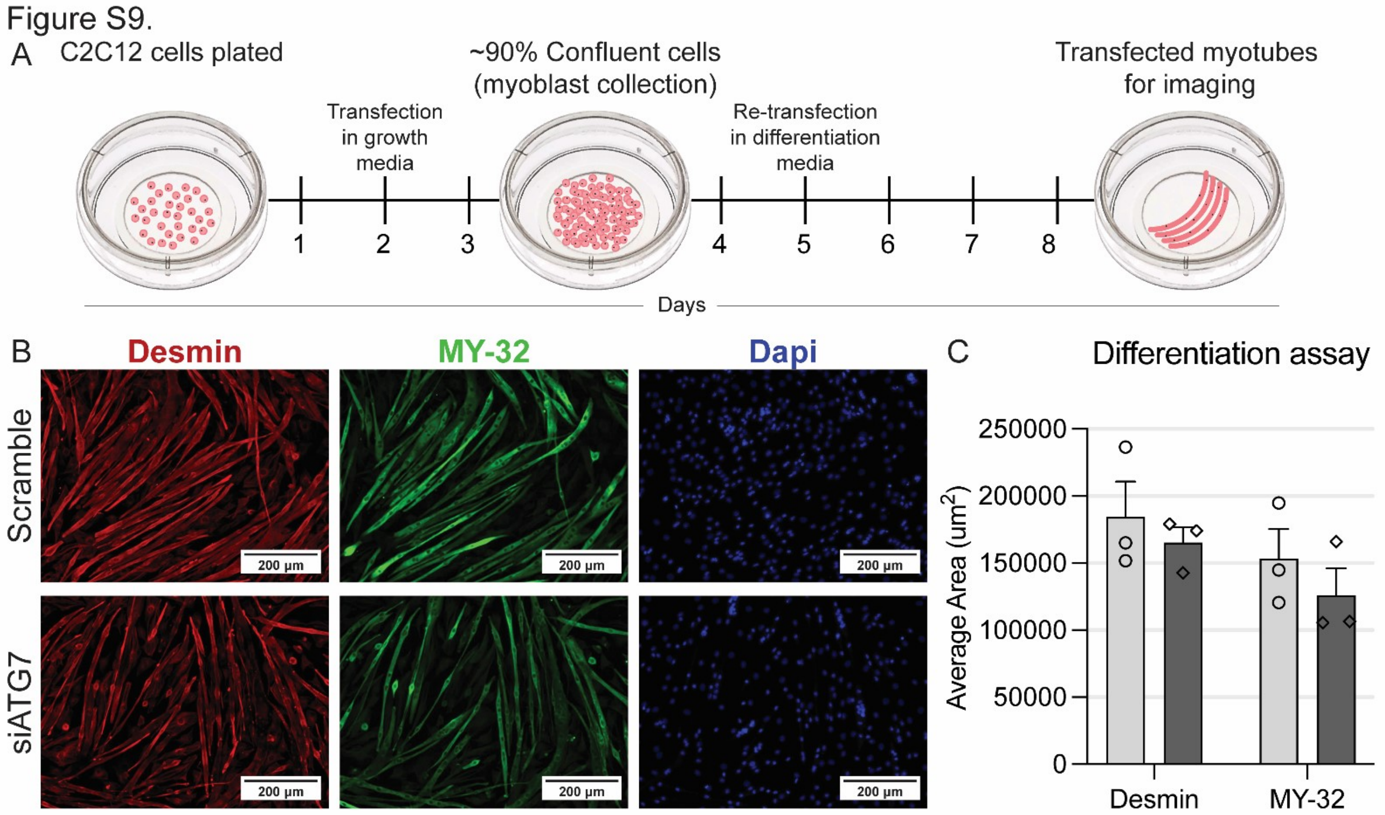

S9: **Autophagy and muscle cellular differentiation**. **A**: Diagram of Atg7 siRNA transfection to myoblasts and myotubes. Notice that myotubes received a second round of transfection, which maintained the silencing of Atg7 product during the experimental window. See methods for details. **B**: Autophagy loss of function does not prevent myoblasts transition into myotubes upon exposure to serum poor (2% horse serum-supplemented) media. This transition is an in-vitro surrogate of myotube differentiation, which involves cell division arrest, elongation of myotubes and their fusion, resulting in mature myofibers expressing multiple proteins of terminally differentiated skeletal muscle. The

expression of desmin and My-32 during that transition is similar between control- and Atg7 siRNA transfected cells, indicating that autophagy is dispensable during the process of differentiation. Disaggregated of desmin and My-32 expression in post Atg7 siRNA transfected myotubes. **C**: Quantification of total fiber area using mature muscle cell markers Desmin or MY-32 shows no difference in cell size between control and Atg7 siRNA treated cells.

Supplementary Tables and files:
Table E1: qPCR primers used for this study.
Table E2: RNA transcripts profiles with p values
Table E3: RNA Metascape results
Table E4: Single cell cluster identification from top 10 transcripts.
Table E5: Single cell Pax7+ cluster upregulated genes of interest.
Table E6: Single cell Pax7+ cluster downregulated genes of interest.
Table E7: Antibodies used for this study.
Supplementary uncropped original immunoblots
Supplementary Materials and Methods

Methods:

Animals: Both male and female mice were used for studies and aggregated data is reported for both sexes unless otherwise specified. **1)** *Wild-type C57BL/6* mice obtained from The Jackson Laboratory were used in this study. At 12 weeks of age mice were placed in either a Biospherix chamber with 10% CO_2 (hypercapnia) or room air as controls (normocapnia) for 4 weeks. **2)** *Wild-type FVB/NJ* mice were also obtained from The Jackson Laboratory for use as a validation strain. **3)** *βActin-DsRed* mice were used for transplantation experiments. These mice express red fluorescent protein variant DsRed.MST under the control of the chicken β-actin promoter coupled with the cytomegalovirus (CMV) immediate-early enhancer(54) and were purchased from the Jackson Laboratory (Stock No: 005441). **4)** *GFP-LC3:* Mice expressing GFP (EGFP)-LC3 cassette inserted between the CAG promoter (cytomegalovirus immediate-early (CMVie) enhancer and chicken β-actin promoter) on a C57BL background were purchased from RIKEN Bio-Resource Center, Japan (#BRC00806). Genotype was determined as previously reported and confirmed by direct visualization of the GFP puncta(59). **5)** *GFP-Pax7*: An animal expressing an IRES-CreERT2 fusion protein downstream the Pax7 stop codon (Jackson Lab, Stock No: 017763) was crossed with a loxP-flanked STOP cassette holding animal (Jackson Lab, Stock No: 007906). The result of that crossing leads to green fluorescence after Cre recombination, which occurs in Pax7-expressing cells. In the present research, we deliberately chose to use mice in early adulthood to avoid the potential confounding effect of aging on the reported myogenic results. Food and water were accessible *ad libitum*, and a 12-hour light/dark cycle was maintained as regulated by our Animal Research Facility (ARF). The time elapsed between animals' withdrawal from the high-CO_2 environment, their euthanasia by cervical dislocation, and sample procurement never exceeded 5 minutes when methodologically feasible.

Venous bicarbonate determination: Values of venous bicarbonate were measured with an i-STAT hand-held blood analyzer (Abbott, Chicago, IL). To do that, animals were restrained, and blood was

obtained using a submandibular bleed, and collected in 100 µL EDTA-coated Microvette capillary collection tubes (Sarstedt).

Food intake: Food intake of normocapnic and hypercapnic animals was compared by recording the difference in the mass of food given to single animals for 3 days. When kept in the hypercapnia chamber, food would absorb excess moisture due to increased ambient humidity. To correct for this confounder, a control container of 100g of food was placed in the chamber during the same 3-day period of monitoring. The percentage of weight gain of the "control" food was used to correct the experimental food masses.

Motion detection: Motion detection cages (Ugo Basile, Gemonio, Italy, 47420) were used for quantitative movement monitoring of animals housed in hypercapnic and normocapnic environments for 24 hours, following standard protocols.

Body weight determination: Mice were weighed using a top loading balance (Mettler Toledo, Ohio).

Muscle procurement and muscle mass: After 4 weeks of normocapnia or hypercapnia the animal was euthanized, immediately after animal euthanasia, leg skin was dissected with tweezers and muscles exposed. Under real time magnification using an illuminated magnifier (Omano, China), muscles were dissected individually, cutting first the distal tendon, and gently removing the entire muscle using dissecting scissors (Roboz RS-5840). After the proximal tendon was severed, the muscle was put in contact with a gauze under magnification to eliminate remaining blood, fat, and if needed also remaining tendon using scissors. Muscle was then weighed using an analytical balance (Sartorius Entris, Germany). Mass determination was performed on the tibialis anterior (TA) muscle. Extensor digitorum longus (EDL) muscles were used for histology, immunofluorescence and contractility analysis.

Muscle histology: After 4 weeks in normocapnic or hypercapnic environments, mice were euthanized, and freshly isolated muscles were chilled on saline-moistened gauze in a 60-mm culture dish on ice. A metal cup containing isopentane was cooled in liquid nitrogen until crystals of isopentane formed at the bottom of the cup. Muscles were transferred to pre-cooled Tissue-Tek embedding cassettes (EMS, Hatfield, PA; 62520), which were dropped into the cooled isopentane, submerging the muscle for 1 min. Muscle samples were then drained and dried on gauze pads at -20°C to remove all isopentane. Frozen muscles were adhered to the sample stage using a small amount of Tissue-Tek optimal cutting temperature (OCT) compound (EMS, Hatfield, PA, 62550) and 10-micron sections were taken for analysis using a Leica CM1860 Cryostat (Wetzlar, Germany).

Muscle immunofluorescence: Muscle sections were fixed for 15 min in acetone at -20°C and then left at room temperature to dry for 30 min. Blocking was performed using mouse-on-mouse blocking reagent (Vector Laboratories, Burlingame, CA) for 1 h at room temperature. Sections were then incubated for 2 hours at room temperature or overnight at 4°C with the indicated primary antibodies. Three 5-minute washes were then performed with PBS. Then, secondary antibodies from Jackson ImmunoResearch Laboratories were added, all at 1:250, and incubated for 1 hour at room temperature. Three washes were then performed with PBS. Samples were mounted with Ibidi mounting medium (Martinsried, Germany). Images were captured on the same day using Cytation 5 (Agilent/BioTek) or confocal microscopy (Leica SPE).

Muscle myofiber cross-sectional area: Muscle sections were stained with anti-laminin and imaged at 10X magnification. CellProfiler software (Broad Institute, Cambridge, MA) was used to measure myofiber cross sectional area in an unbiased manner. Output muscle traces were reviewed to assure accuracy of measurement of the software pipeline.

Grip strength test: Animal limb grip strength was determined using a Grip Strength Meter (Ugo Basile, Germonio, Italy, 47200). The mouse was held by the tail and placed on the waffle grasping grid. Pressure was steadily applied to the mouse's tail until failure and release from the grid occurred, at which time the peak force was measured. This procedure was repeated 5 times per animal with one minute resting periods.

Isolated muscle contractility: Absolute force determination: Extensor digitorum longus (EDL) muscles were surgically isolated from the mouse by carefully tying a suture around the tendon at each end and cutting the tendons to release the muscle. Special care was taken not to stretch or damage the muscle integrity, as previously established. Once removed, the analyzed muscle was equilibrated for 15 min in ice-cold Ringer's solution supplemented with 5.5 mM glucose, adjusted to a pH between 7.4 and 8.0, and slowly bubbled with carbogen. The muscle was then suspended between the isometric force transducer (Harvard Apparatus, Holliston, MA) and the platinum-stimulating electrode tissue support (Radnoti, Covina, CA; 160152), lowered into the 25-mL tissue bath (Radnoti, Covina, CA; 166026) containing the same solution, also bubbled slowly with carbogen, but this time at room temperature; and allowed to equilibrate for an additional 15 min. Muscle tension was escalated until baseline tension started to increase. A single 1-Hz, 40-V stimulus was delivered with a Grass S-88 electrical stimulator, and the peak contraction was recorded. After a 30s rest, voltage was increased by 10 V and delivered again, recording peak contraction. This process was repeated until no additional increase in the peak contraction force was observed. The optimal length of the muscle was then determined by slowly increasing the muscle tension and delivering a single, maximal stimulus, as previously determined above, while recording the peak force. After a 30s rest, tension was slightly increased, and another stimulus was delivered while recording peak contraction. This process was repeated until maximal peak contraction force was achieved. Subsequent stimuli were delivered at 1, 10, 20, 30, 50, 80, 100, and 120 Hz, while recording the peak force at each point and allowed for 1

minute of rest between each stimulus. Specific force determination: After the final stimulus was recorded, the muscle mass was determined. With that information, the following equation was used to obtain the specific force at each point: Specific Force (N/cm2) = peak contraction (kg) × 9.8 (m/s2) × Optimal Length (cm) × 1.056 (g/cm3)/ muscle mass (g). From the single twitch contraction (1 Hz) dT/dtmax (contraction time) and −dT/dtmax (relaxation time) was determined. The slope (in units of g/s) was calculated from raw contraction data at each point from the baseline to the maximum value (that is dT/dtmax) and then the most negative one (that is −dT/dtmax).

***Induction of muscle injury*:** Mice were anesthetized with isoflurane and hair was removed from the hind legs using Nair®. The right tibialis anterior (TA) muscle of the hind leg was injected with 50uL of sterile saline while the left TA muscle was injected with 50uL of 10uM cardiotoxin in sterile saline as previously established. Mice were left to recover in their cage while closely monitored. Twenty-one days after the initial injury, a second injury was performed using the same protocol. Four to twelve days after the second injury procedure, the TA muscles were collected and frozen histologically.

Hematoxylin and eosin staining: Slides were fixed (10% formalin and 5% acetic acid), washed in 70% ethanol, 90% ethanol, running water, and deionized water prior to being incubated in hematoxylin (Richard Allen Scientific, San Diego, CA #7211) for 2 minutes. Slides were then rinsed in running water, incubated in clarifier (Richard Allen Scientific, San Diego, CA #7401), washed, and then bluing reagent (Richard Allen Scientific, San Diego, CA #7301) was applied for one minute. Slides were washed again in running water and 95% ethanol prior to incubation in Eosin Y (Richard Allen Scientific, San Diego, CA #7111) for 35 seconds. Slides were further washed with 95% and 100% ethanol and finally incubated in Xylenes (Honeywell, Morris Plains, NJ #534056) for 2 minutes. Slides were dried for 2 minutes prior to mounting (Richard Allen Scientific, San Diego, CA #4112).

***Satellite cell isolation (MACS)*:** Skeletal muscles were collected from the hind legs of the mice. Cellular dissociation was performed, and mouse skeletal muscle satellite cells were first enriched using a Mouse Satellite Cell Isolation Kit (MACS Miltenyi Biotech,130104268) and then selected for using anti-integrin α-7 microbeads (MACS Miltenyi Biotech,130104261) to ensure population purity as previously established.

***Flow-sorted Pax7-GFP satellite cells (FACS)*:** Mice bred with an inducible Pax7-GFP reporter (Pax7-Cre$^{+/-}$Lox-GFP$^{+/+}$) were placed in the 10% CO_2 chamber as described above. Age and sex matched control mice with the same genotype were placed in room air. Mice were induced with 500 mg/kg tamoxifen chow (Envigo, TD.130857) for two consecutive weeks during the first two weeks of the four-week period. Animals were euthanized, hind leg muscles were collected and dissociated, as previously described. Filtered cells were resuspended in 300uL of buffer (3mM EDTA, 5% FBS). The cells were then washed with buffer one additional time and filtered for sorting with our BD FACSAriaTM II. Correct gating was established with WT non-fluorescent control cells. Fluorescent Pax7-GFP-positive cells were collected, centrifuged, and buffer was removed before plating.

***EdU proliferation assay*:** Cells, isolated as previously described from normocapnic or hypercapnic animals, were plated on 20mm insert glass bottom plates (MatTek, P35G1.520C) with growth media containing 0.05 mM EdU. The media was changed carefully every 16 hours until the cells were fixed at 40 hours using 4% paraformaldehyde for 30 minutes at 4°C. EdU incorporation was visualized with a Click-iT kit (Thermo Fisher Scientific, C10337) following the manufacturer’s instructions and as previously established. Ex-vivo rapamycin treatment was performed similarly; however, cells were treated with 10 mM of rapamycin or vehicle for 24 hours before fixation. In-vivo rapamycin experiments were designed similarly, however, animals in normocapnic or hypercapnic environments were injected with 10mg/kg/day rapamycin for 14 days until collection.

Pax7-GFP injury recovery assay: 12-week-old mice, bred with an inducible Pax7-GFP reporter ($Pax7\text{-}Cre^{+/-}Lox\text{-}GFP^{+/+}$) were placed in the 10% CO_2 chamber as described above. Age and sex matched control mice with the same genotype were placed in room air. Mice were induced with 500 mg/kg tamoxifen chow (Envigo, TD.130857) during the first two weeks of the four-week period. Mice were anesthetized and muscle injury was performed as described previously. Mice were euthanized after 21 days, and TA muscles were procured histologically. The GFP mean fluorescence intensity of the entire muscle section was determined using a Cytation 5 (Agilent/BioTek). As hypercapnia has been associated with increased spontaneous injury-repair events, the uninjured counter lateral TA muscle was used as a baseline fluorescence measurement.

Myoblasts transplantation: One day before the transplantation, injury was performed on recipient animals by removing the hair from the hind legs and injecting each of the animal's TA muscle with 25uL of 10uM cardiotoxin. Simultaneously, animals began receiving 15mg/kg Cyclosporine A by subcutaneous injection (Novartis, East Hanover, NJ) which was continued every day until the muscle collection date and provided to increase the allotransplanted cells viability. Integrin α-7 enriched satellite cells were collected from donor mice as previously described, and 50,000 cells were immediately injected into each TA of the recipient mouse: one TA received cells from an experimental donor while the contralateral leg, used as an internal control, received cells from a control animal. Two weeks after the transplant, TA muscles were collected and histologically frozen. Fresh frozen sections were obtained, and images were immediately acquired using a Leica fluorescent microscope and total numbers of auto-fluorescent red cells were counted. Rapamycin experiments were conducted similarly however, donor animals in normocapnic or hypercapnic environments were injected with 10mg/kg/day rapamycin for 14 days until collection.

RNA extraction, cDNA synthesis, and quantitative RT-PCR: RNA from cells was extracted using standard TRIzol extraction. cDNA was synthesized using Quantitect Reverse Transcriptase Kit

(Qiagen) following manufacturer's protocol. Quantitative RT-PCR was performed using iTaq Universal SYBR Green Supermix (Bio-Rad) on a CFX96 Real-time PCR detection system (Bio-Rad). Each sample was run in triplicate, and relative expression levels of transcripts of interest were calculated using the comparative Ct (ΔΔCt) method with glyceraldehyde-3-phosphate dehydrogenase (GAPDH) as a housekeeping gene. Primers were purchased from Integrated DNA Technologies (IDT, IA).

Post-injury Pax7-GFP cell RNA-sequencing: Induced Pax7-GFP reporter normocapnia or hypercapnia mice were housed as described previously. Muscle injury was produced by injecting 10uM cardiotoxin (Millipore Sigma, 217503) in 10uL aliquots along the muscle body - each tibialis anterior (TA) muscle was injected with 40uL total, and each gastrocnemius (GN) muscle was injected with 80uL total. Three days after the injury, all hind leg muscles were collected, and cells were flow-sorted as previously described. RNA was isolated using TRIzol with an added step of DNA digestion. RNA quantity was determined using a Qubit Flex Fluorometer (Thermo Fisher Scientific, San Jose, CA). Library preparation was performed using an Ion Chef System (Thermo Fisher Scientific, San Jose, CA) followed by sequencing using an Ion GeneStudio S5 Plus System (Thermo Fisher Scientific, San Jose, CA) both following manufacturer's suggested protocols for the Ion AmpliSeq Transcriptome Mouse Gene Expression Kit (Thermo Fisher Scientific, San Jose, CA). Differential expressions were determined using the Transcriptome Analysis Console (TAC) and pathway enrichments were performed using Metascape.

Single-cell RNA sequencing and data analysis: Single-cell RNA sequencing was conducted commercially by GENEWIZ on a 10x Genomics droplet-based platform following manufactures standard protocols for fixed cell Chromium Fixed RNA Profiling. Data were analyzed using Seurat package (Version 5.0.1) in *R*Studio software (Version 4.3.3). We processed single-cell RNA-seq data using Seurat in RStudio, filtering out cells with unique molecular identifier counts below 200. We

normalized gene expression data using LogNormalize in Seurat and identified the 2,000 most variable features using the vst method with FindVariableFeatures. The top 10 most variable genes were selected for labeling on a variable feature plot. All gene expressions were scaled to Z-scores with ScaleData. PCA was then conducted using the variable features, with visualization of the PCA results including cell and feature loadings, elbow plot, and dimension plots for the first 15 dimensions. We reduced dimensionality using UMAP on the top 9 principal components to create a UMAP plot for visualization. This data was visualized in two dimensions, clustered (FindClusters), and differentially expressed genes (DEGs) were pinpointed (FindMarkers). Cell types for the control samples were determined using DEGs and corresponding markers. Gene ontology of DEGs was analyzed with GSEA, setting thresholds at fold change over 0.5 and adjusted *P*-value below 0.05.

LC3-GFP autophagy assay: Mice 12 weeks old, bred with a constitutively active LC3-GFP reporter (LC3-GFP$^{+/+}$) were placed in the 10% CO_2 chamber as described above. Age and sex matched control mice with the same genotype were placed in room air. Satellite cells were isolated from the hind legs of these mice, as previously described. Cells were plated and treated with bafilomycin for 4 hours before fixation and nuclear staining. Mean fluorescence intensity (MFI) was measured automatically using a Cytation5 cell imager (Biotek, Winooski VT). Rapamycin experiments were designed similarly however, animals were injected with 10mg/kg/day rapamycin for 14 days until collection. **Primary satellite cells and myoblasts**: Freshly column-isolated satellite cells were plated on laminin-521 (Thermo Fisher Scientific, San Jose, CA, CB40221) coated plates and allowed to seed for 24 hours before switching to buffered normo- or hypercapnia media as previously described. Each plate used for unique experimental conditions was contributed by an individual animal. After 4 days of normo or hypercapnia exposure, cells were treated with 10 μM of rapamycin or vehicle for 24 hours.

Western blotting: Once appropriate for each experiment, media was aspirated from the plates, cells washed with PBS, and 200 µL of Muscle Lysis buffer pH 7.6 with complete EDTA-free Protease Inhibitor Cocktail tablet (Sigma Aldrich, St. Louis, MO) was added. Plates were scraped and cell lysate was collected. Samples were prepared for western blot using Bio-Rad 4X Laemmli Buffer, and protein amounts were determined and normalized by BSA Assay (Thermo Scientific Pierce Protein Biology Products, Waltham, MA). Experiments were run using either a 15 or 10 well 4-20% gradient polyacrylamide gel or a 15 or 10 well 10% polyacrylamide gel (Bio-Rad, Hercules, CA) for 45 to 55 minutes at 150 volts or 25 mA. After running, samples were transferred to a 0.45 um Nitrocellulose Membrane (Bio-Rad, Hercules, CA) using either wet transfer for 40 to 50 minutes at 100 volts or a semi-dry transfer for 20 to 30 minutes at 10 to 15 volts. Membranes were blocked with 5% dry milk in 1X TBST for 1 hour at room temperature (RT). Primary antibodies were incubated at a 1:500 or a 1:1000 in 1% milk in 1X TBST with appropriate antibody overnight at 4℃. Membranes were then washed 3 times for 10 minutes in 1X TBST at room temperature, and then HRP conjugated secondary antibodies (Jackson ImmunoResearch Laboratories, West Gove, PA, #111-035-144 and #115-035-146) were incubated at a 1:1000 to 1:2000 ratio in 1X Clear Milk Blocking Buffer (CMBB) in 1X TBST (Thermo Scientific, Waltham, MA); for 1 to 2 hours. Blots were washed 3 times for 15 minutes in 1X TBST at room temperature. Chemiluminescent pictures were taken using the ChemiDoc MP Imaging System (Bio-Rad, Hercules, CA, #734BR3626) by activating the HRP with ECL reagents (Bio-Rad, Hercules, CA). Blots were quantified using Bio-Rad Image Lab software. Experiments conducted to immunodetect LC3 (Cell Signaling Danvers, MA, #4108) were run on gradient gels with a shorter transfer time of 40 to 42 minutes. ACC (Cell Signaling, Danvers, MA, #3662 and #3661) and Ulk1 (Cell Signaling, Danvers, MA, #8054, #5869, and #6888) blots were run on 10% gels with a transfer time of 50 to 55 minutes. mTOR (Cell Signaling, Danvers, MA#2983 and #5536) blots were run on 4-20% gels with a transfer time of 50 to 55 minutes. AMPK (Cell Signaling, Danvers, MA, #2532 and #2535) blots

were run on a 10% gel with a transfer time of 45 to 50 minutes. ***In-vitro*** **myoblast model: C2C12 cells**: 60-millimeter or 6- well culture dishes (Celltreat, Pepperell, MA) were inoculated with C2C12 cells (ATCC, Manassas, Virginia) at a seeding density of 2 x 10^3 cells per cm^2 in growth media containing Dulbecco's Modified Eagle's Medium (DMEM) High Glucose (Corning, Glendale, AZ), 10% Fetal Bovine Serum (FBS, Sigma-Aldrich, St. Louis, MO), and 1% Penicillin and Streptomycin (P/S). Growth media was aspirated after 24 hours and buffered normocapnia or hypercapnia media, preconditioned for at least two hours, was added to plates. Normocapnia media contained DMEM growth media, Hams-F12 (Corning, Corning, NY), 0.5 M 3-(N-morpholino) propanesulfonic acid (MOPS) pH 7.2 (Sigma-Aldrich, St. Louis, MO), and 0.5 M Trizma base (TRIS) pH 7.0 (Sigma-Aldrich, St. Louis, MO). Hypercapnia media contained DMEM growth media, Hams-F12, 0.5 M MOPS pH 12.0, and 0.5 M TRIS pH 10.0. Plates were then incubated in buffered media for 4 days in respective normocapnia (5% CO_2) or hypercapnia (20% CO_2) incubators (VWR, Radnor, PA). Differentiation media was made similarly as described above replacing 10% fetal bovine serum with 2% horse serum to stimulate differentiation into myotubes. For rapamycin treated experiments, cells were treated with 10μM of Rapamycin or vehicle for 24 hours before utilizing cells for downstream analysis. For bafilomycin treated experiments, cells were treated with 10 μM or vehicle for 4 hours before utilizing cells for downstream analysis. For MG-132 treated experiments, cells were treated with 5 μM for 24 hours before utilizing cells for downstream analysis.

In-vitro **C2C12 Atg7 siRNA model:** 6-well culture dishes were inoculated with C2C12 cells (ATCC, Manassas, Virginia) at a seeding density of 3x10^3 cells per cm^2 in growth media. Cells were transfected using lipofectamine 3000 and 20nM of Atg7 siRNA or 20nM Scramble siRNA (Santa Cruz Biotechnologies, Dallas, TX, sc-41448) following manufacturer's suggested protocols. Plates were then incubated for 4 days. For rapamycin treated experiments, cells were treated with 10μM of Rapamycin or vehicle for 24 hours before lysing cells for downstream analysis. For bafilomycin treated experiments,

cells were treated with 10 μM or vehicle for 4 hours before utilizing cells for downstream analysis. For differentiation assay, myoblasts were re-transfected following the same protocol as previously mentioned at 90% confluency while simultaneously switching to differentiation media(7). For differentiation staining, cells were fixed using 4% PFA (Thermo Fisher Scientific, San Jose, CA), cell membranes were permeabilized with 0.1% TritonX-100 (Sigma-Aldrich, St. Louis, MO), and blocked with 5% Goat Serum (Vector Laboratories, Burlingame, CA #S-1000-20)., 2% BSA, and 1:40 MOM (Vector Laboratories, Burlingame, CA #MKB-2213-1). Primary antibodies, Desmin (Abcam, Cambridge, UK, #ab15200) and MY-32 (Sigma-Aldrich, St. Louis, MO, #M1570), were incubated in a 1:300 and 1:200 dilution overnight. Plates were washed with PBS and secondary antibodies were incubated in a 1:250 dilution using fluorescent Alexa Fluor 594 anti-rabbit and Alexa Fluor 488 anti-mouse antibodies (Life Technologies Co, Eugene, OR, #Z25307 and #Z25302). Plates were washed and incubated with DAPI. Images were taken using the Cytation 5 (Agilent Technologies, Santa Clara, CA) at a 10x magnification.

Immunoprecipitation (IP): C2C12 cells were treated with MG-132 at 5 μM for 24 hours. Cells were collected and protein concentrations were normalized at 150 μg of protein using a Bradford assay (Thermo Scientific Pierce Protein Biology Products, Waltham, MA). AMPKα1 antibody (Abcam, Cambridge, UK #ab32047) was biotinylated with a Type B Biotinylation Kit (Abcam Cambridge, UK #ab201796) following manufactures protocol. For the IP, 200 μg of streptavidin magnetic beads (New England Biolabs Ipswich, MA #S1420S were washed with 1X TBST twice and then incubated in lysis buffer with 7.6 μg of biotinylated AMPKα1 antibody for 30 minutes at 4℃ rotating top-over bottom. Beads were magnetized using a magnetic stand (Bio-Rad, Hercules, CA #1614916) and supernatant was discarded. Beads were washed twice with 1X TBST and incubated with 150 μg of protein lysate in a total volume of 200 μL overnight at 4℃ rotating top-over bottom. Beads were magnetized using a

magnetic stand, supernatant was discarded, and beads were washed three times in 1X TBST. Samples were eluted with 40 μL of 4X Laemmli Buffer with BME (Bio-Rad # 1610747 and #1610710) for 10 minutes at 70℃. Beads were removed and elution was transferred to a clean tube and used for western blot analysis.

***Proximity ligation assay (PLA)*:** C2C12 cells were plated at $1.5x10^3$ cells per cm^2 in an 8 well chamber slide with growth media (IBIDI, Munich, Germany #80841). Growth media was aspirated after 24 hours and buffered normocapnia or hypercapnia media, preconditioned for at least two hours, was added to plates, then cells were incubated for 4 days. Respective wells were treated with 10μM Rapamycin or vehicle for 24 hours. Cells were fixed using 4% PFA, permeabilized for 10 minutes at RT with 0.1% TritonX-100, and blocked with 5% Goat Serum, 2% BSA, and 1:40 MOM for 1 hour at 37℃. Primary antibodies AMPKα1 and ULK1 were incubated overnight at 4℃ at a concentration of 5.0 μg/mL (Novus Biologicals, Centennial, CO #NBP2-22127) and 3.13 μg/mL (Cell Signaling, Danvers, MA, #8054), respectively. Cells were washed with Wash Buffer A (Sigma-Aldrich, St. Louis, MO, #DUO82046). Chamber walls were removed from slides prior to the addition of PLA probes. Plus and Minus PLA Probes (Sigma-Aldrich, St. Louis, MO #DUO92002 and #DUO92004) were added and incubated at concentrations and time suggested in manufacturer protocol. Ligation and amplification were performed per manufacturers protocol using Duolink® In Situ Detection Reagents Red (Sigma-Aldrich, St. Louis, MO #DUO92008). Cells were stained with DAPI for 30 minutes at RT before coverslips were mounted using VECTASHIELD® mounting medium (Vector Laboratories, Burlingame, CA, #H-1000-10) and sealed using clear nail polish. Slides were imaged using BioTek Cytation5 Cell Imaging (Agilent Technologies, Santa Clara, CA) and quantified using mean fluorescence intensity (MFI) automatically using the Cytation5 cellular detection software.
