# Supplementary material for: Rapamycin improves satellite cells’ autophagy and muscle regeneration during hypercapnia
Source: JCI Insight. 2025 Jan 9;10(1):e182842. doi: 10.1172/jci.insight.182842 (PMC11721297; doi:10.1172/jci.insight.182842)

Primary Cell Baseline

Primary cells in NC or HC no rapamycin

Figure 7D

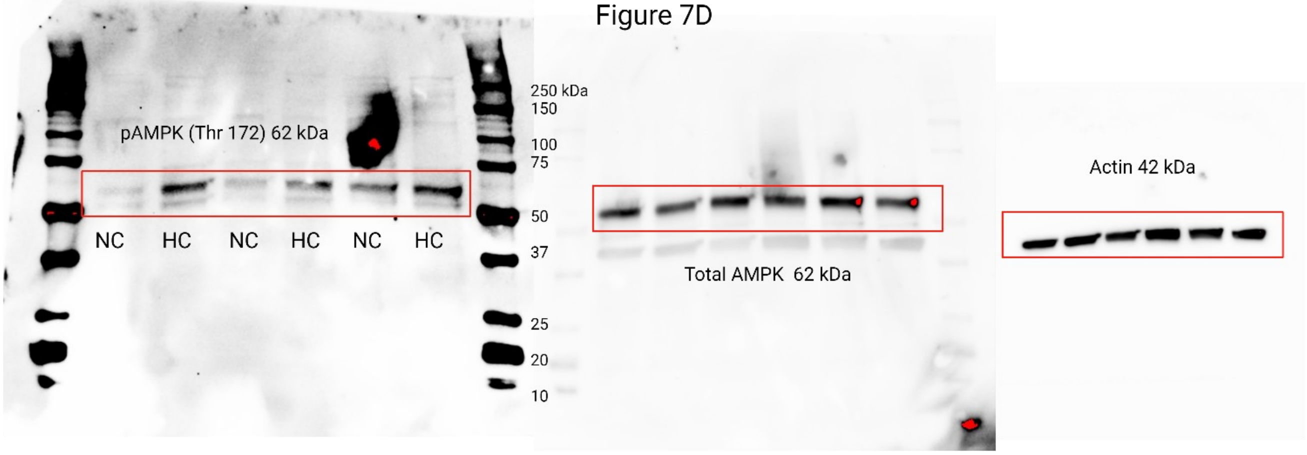

Figure 7D

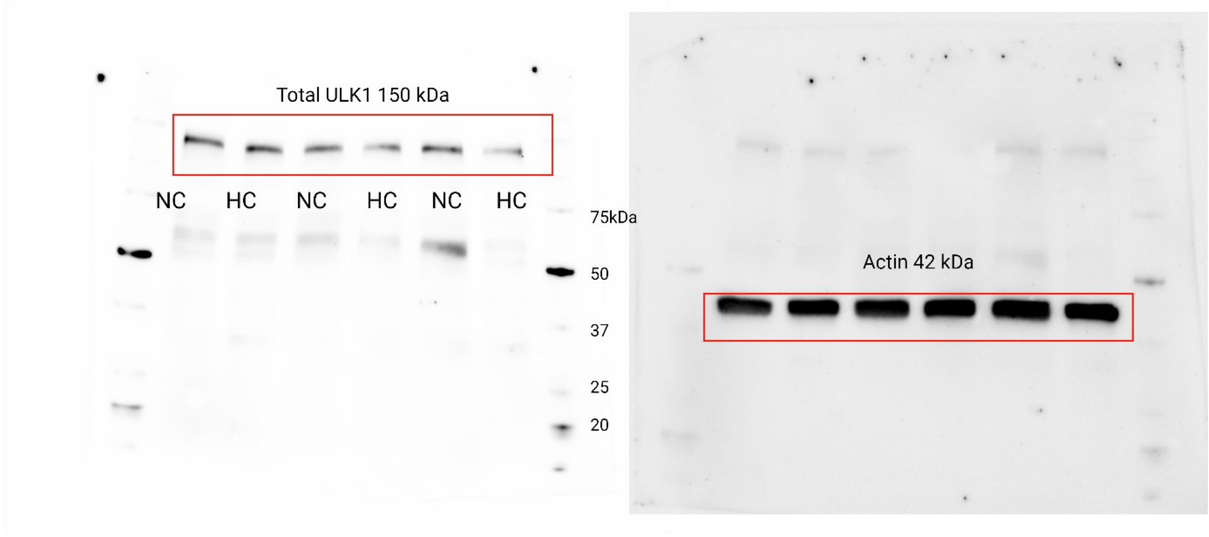

Figure 7D

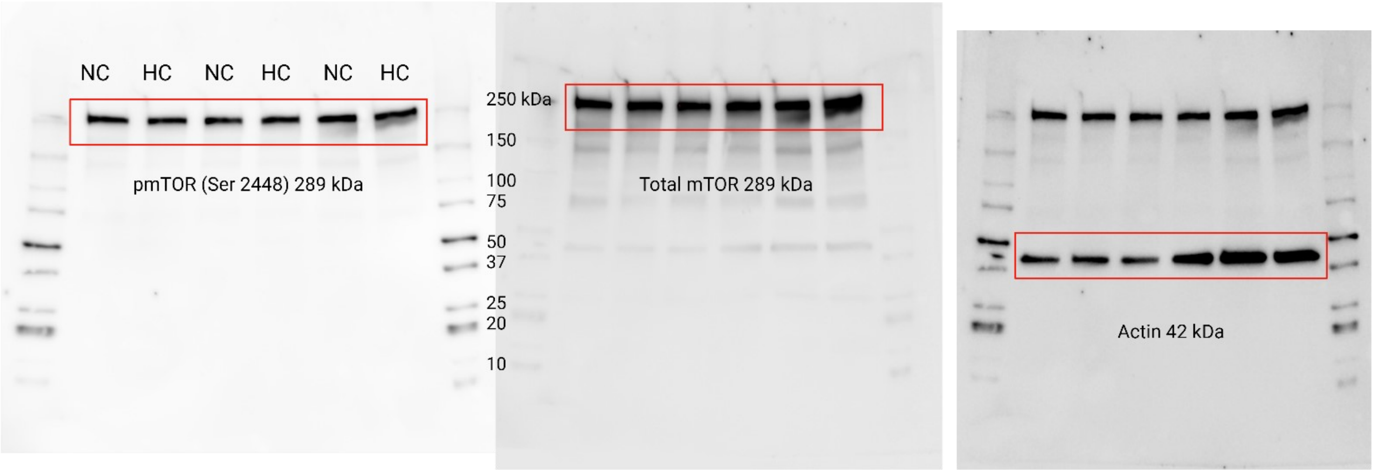
NC
HC
NC
HC
NC
HC
pmTOR (Ser 2448) 289 kDa
250 kDa
150
100
75
50
37
25
20
10
Total mTOR 289 kDa
Actin 42 kDa

Primary cells treated in NC or HC with or without rapamycin

Figure 7E

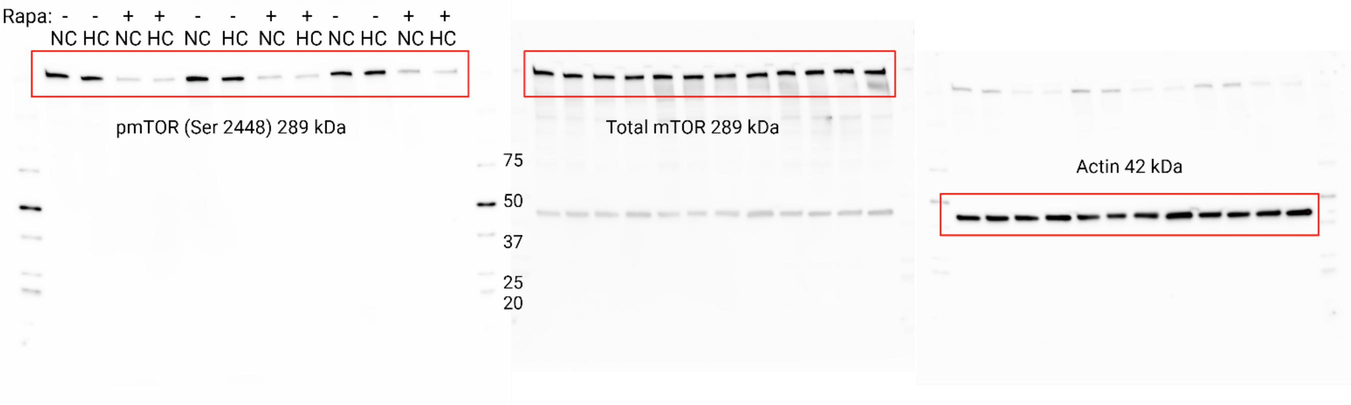

Figure 7E

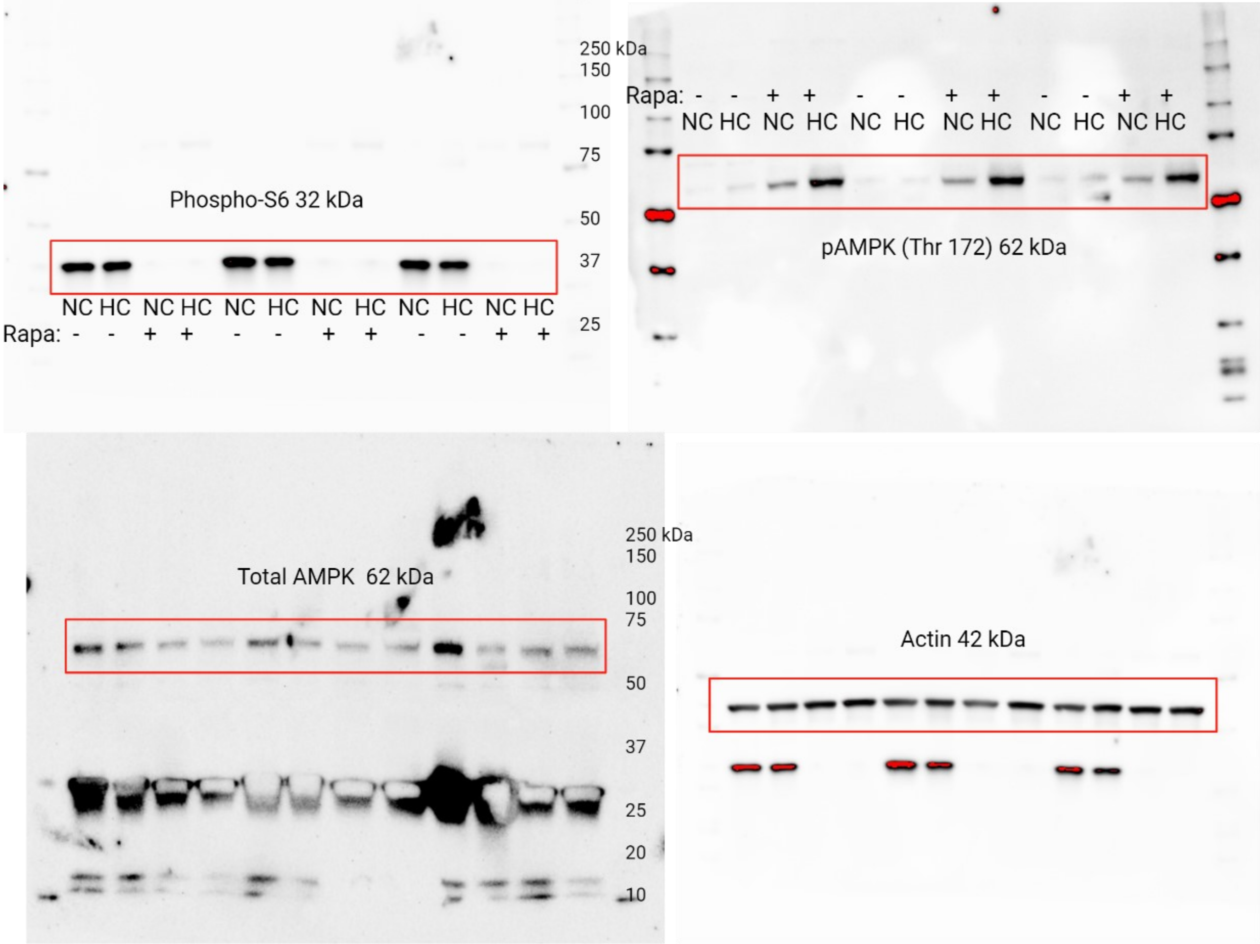

Baseline Autophagy

Figure 9B

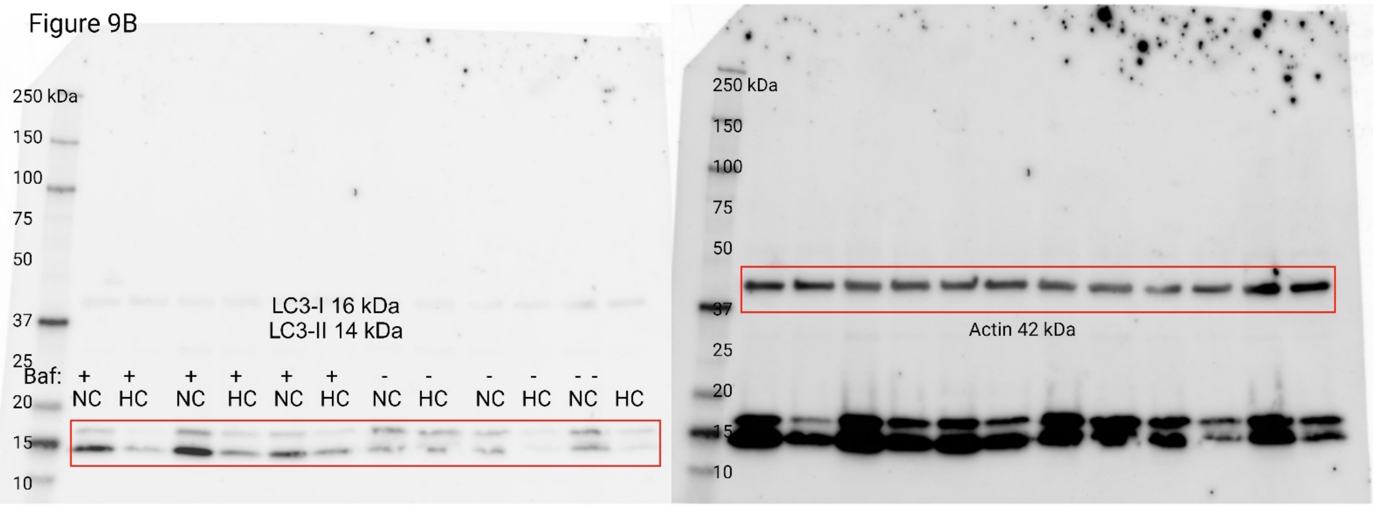

Figure 9C

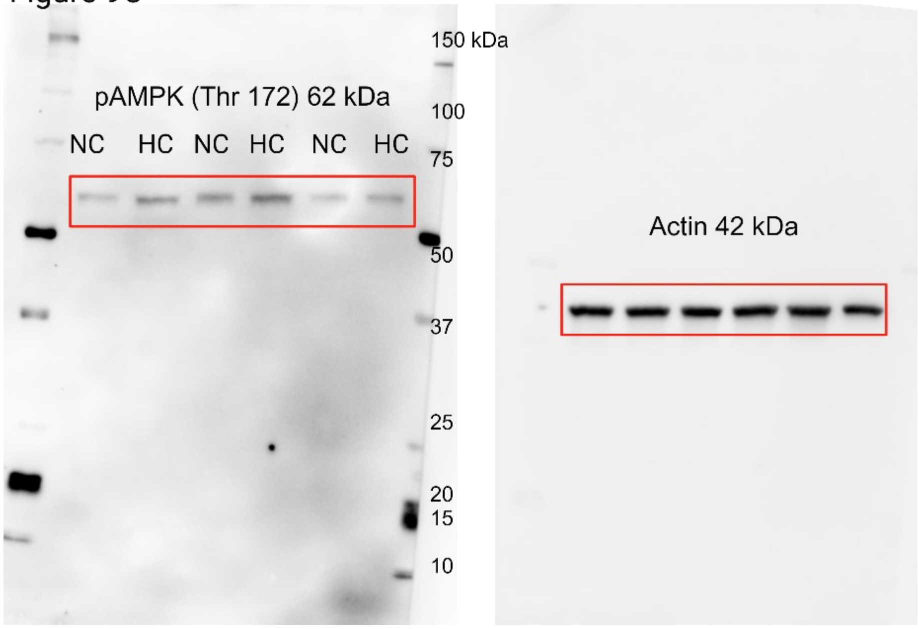

Figure 9C

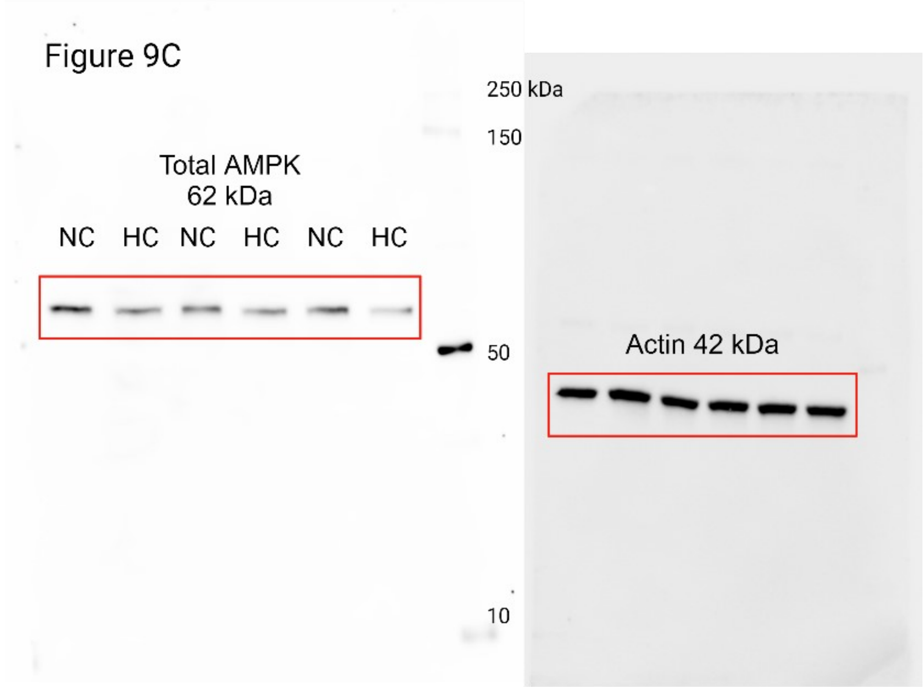

Figure 9C

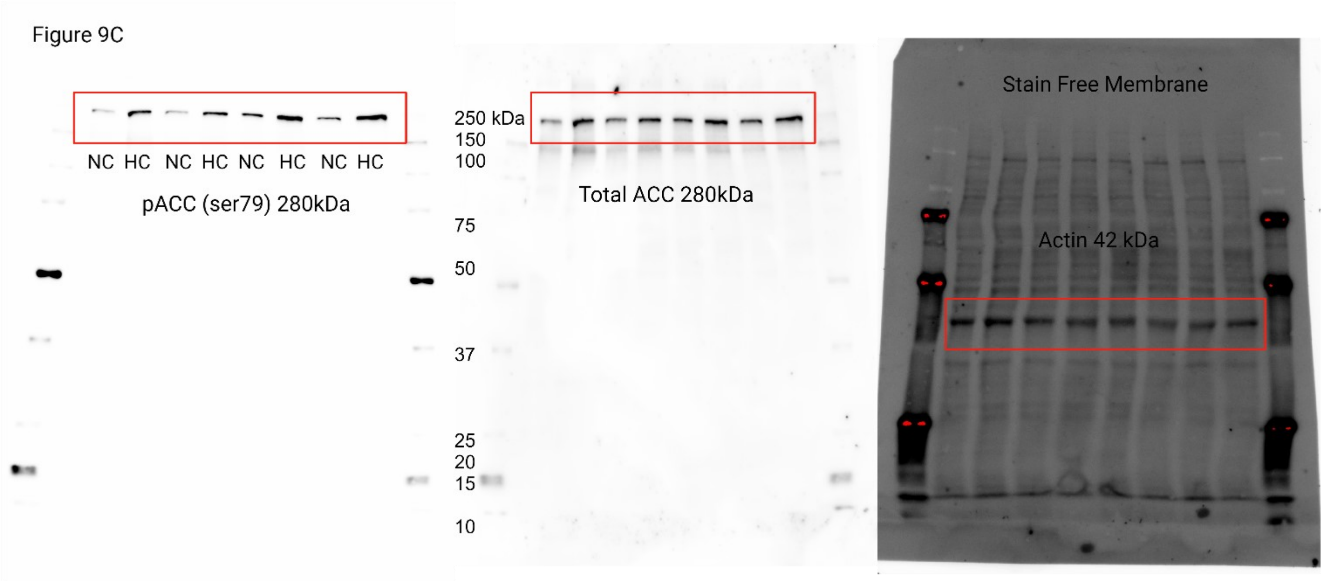

Figure 9C

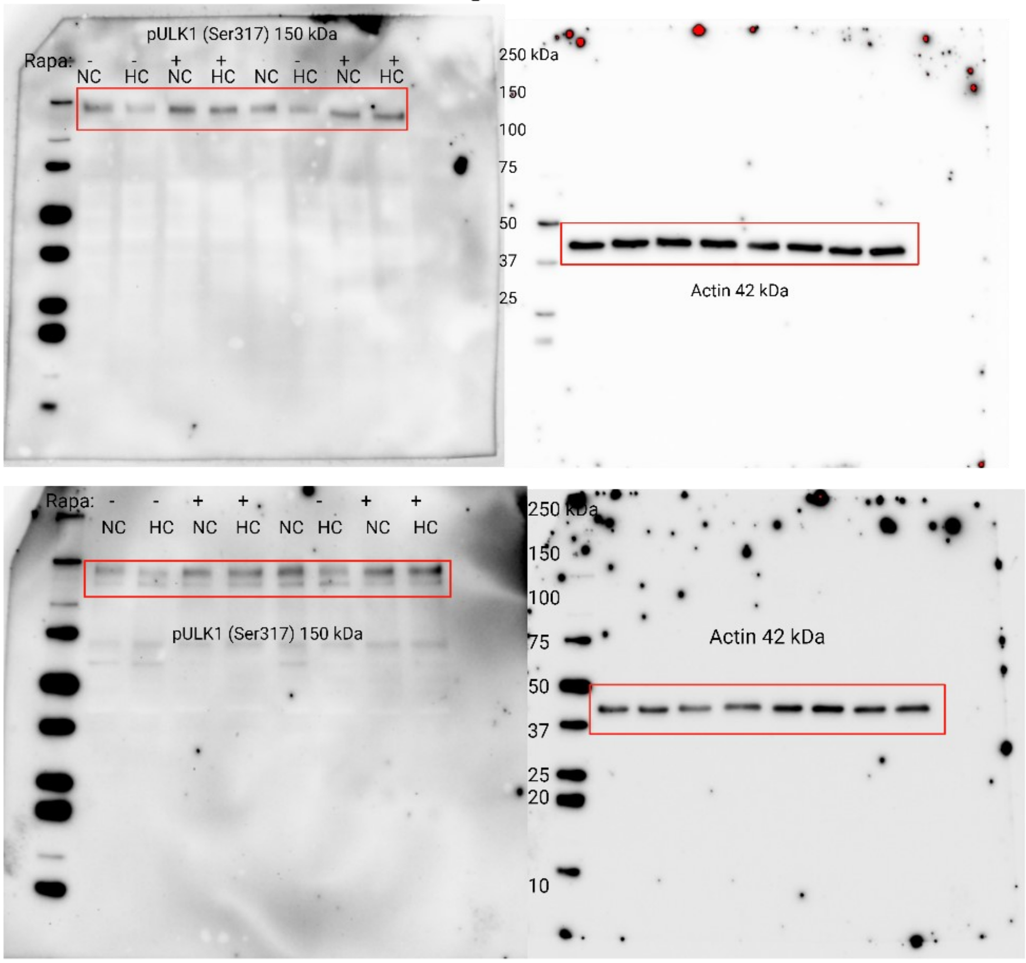
pULK1 (Ser317) 150 kDa
Rapa: - - + + - - + +
NC HC NC HC NC HC NC HC
250 kDa
150
100
75
50
37
25
Actin 42 kDa
Rapa: - - + + - - + +
NC HC NC HC NC HC NC HC
pULK1 (Ser317) 150 kDa
250 kDa
150
100
75
50
37
25
20
10
Actin 42 kDa

Figure 9C

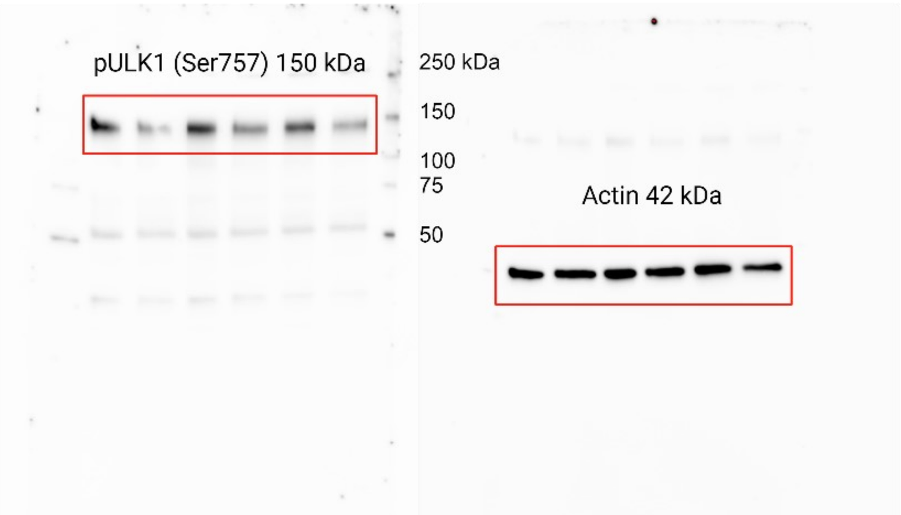

Figure 9C

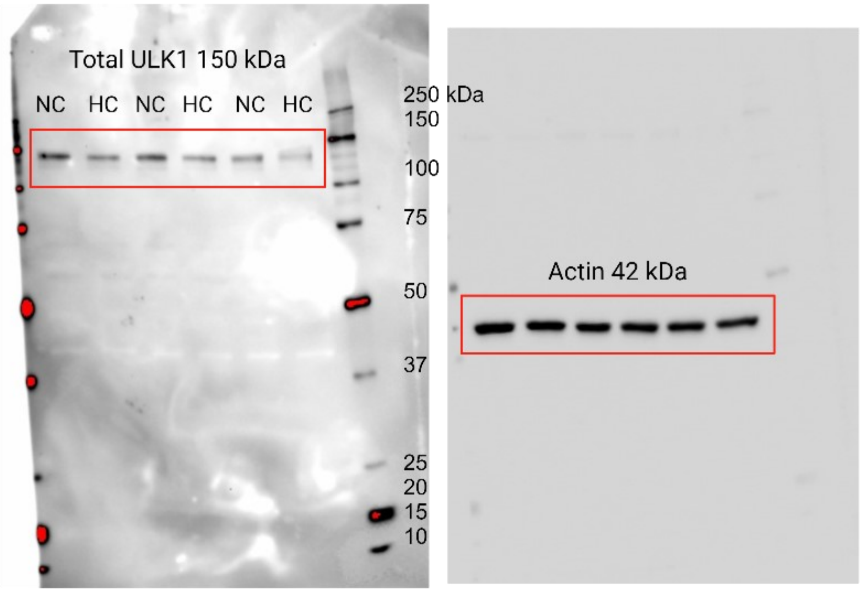

Figure 9C

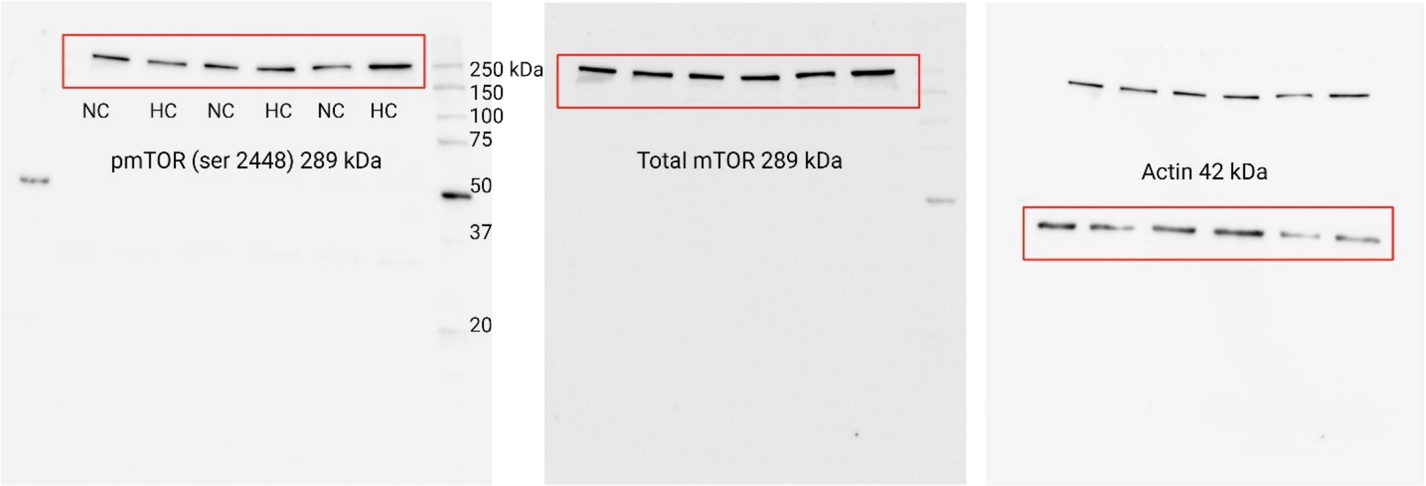

Rapamycin Stimulation

Figure 9F

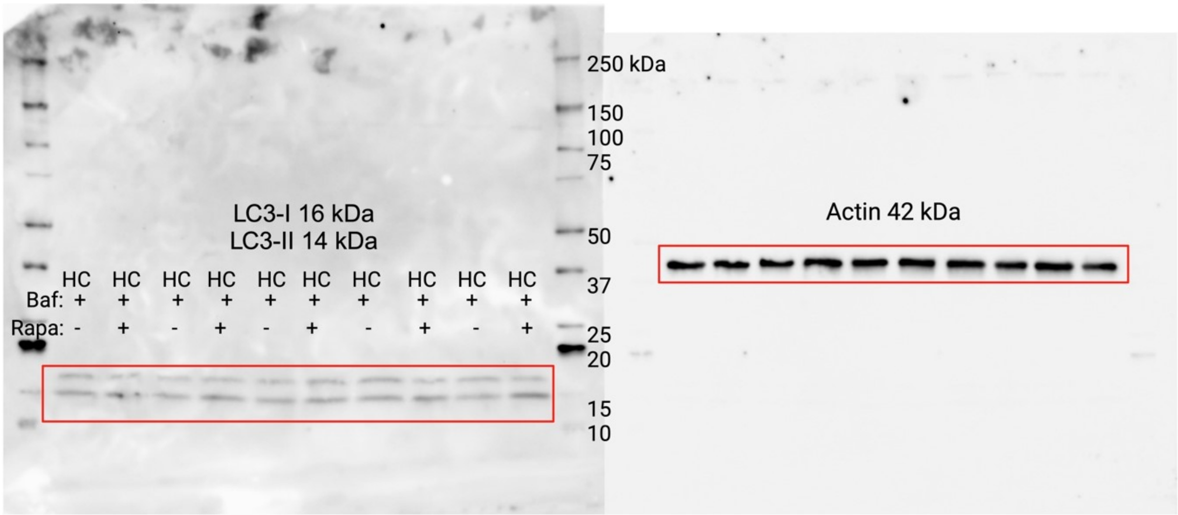

Figure 9H

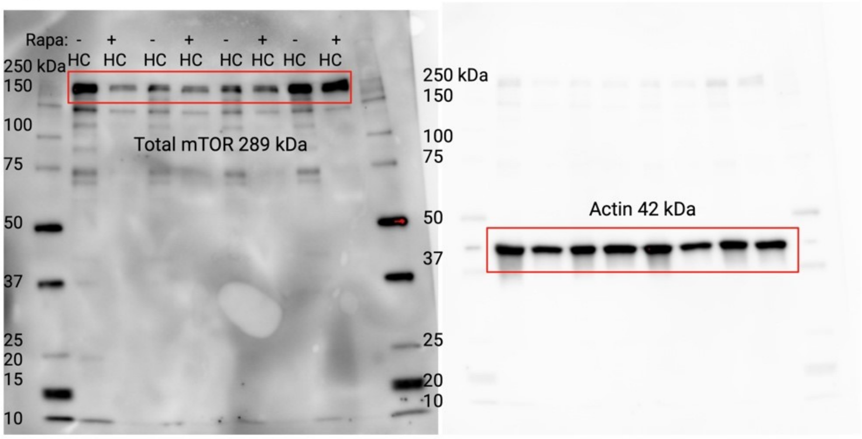

Figure 9H

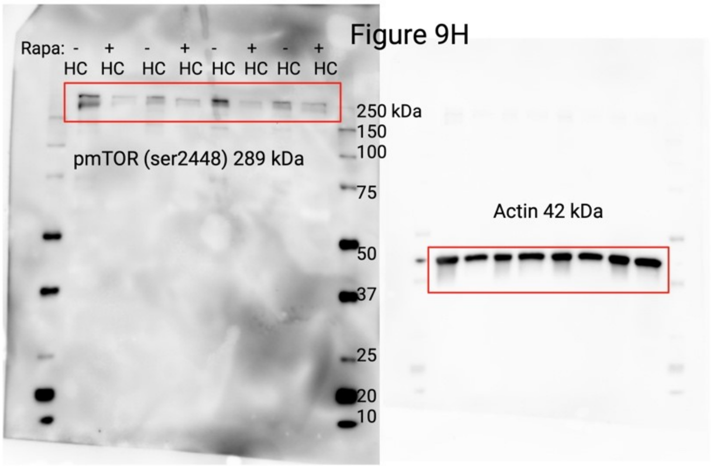

Figure 9H

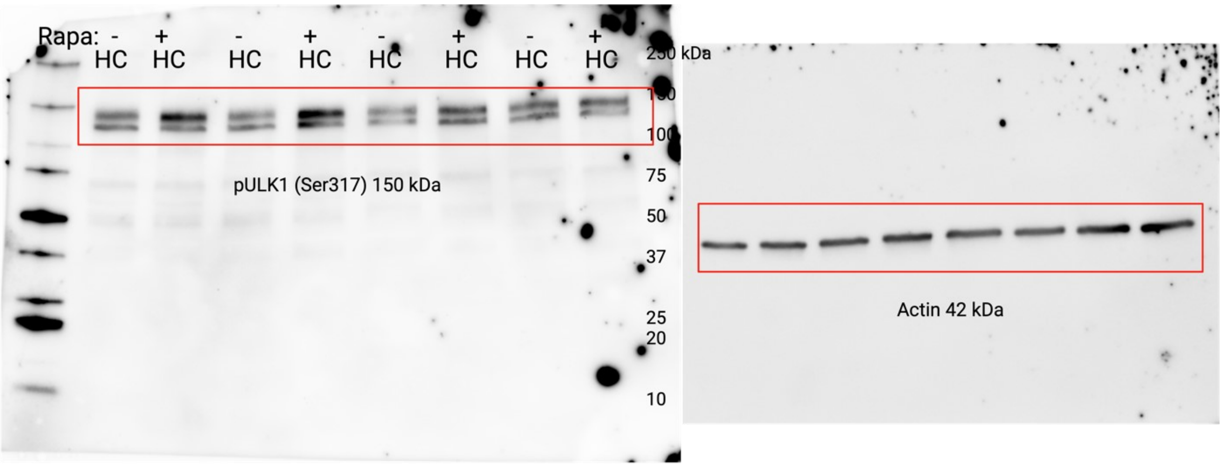

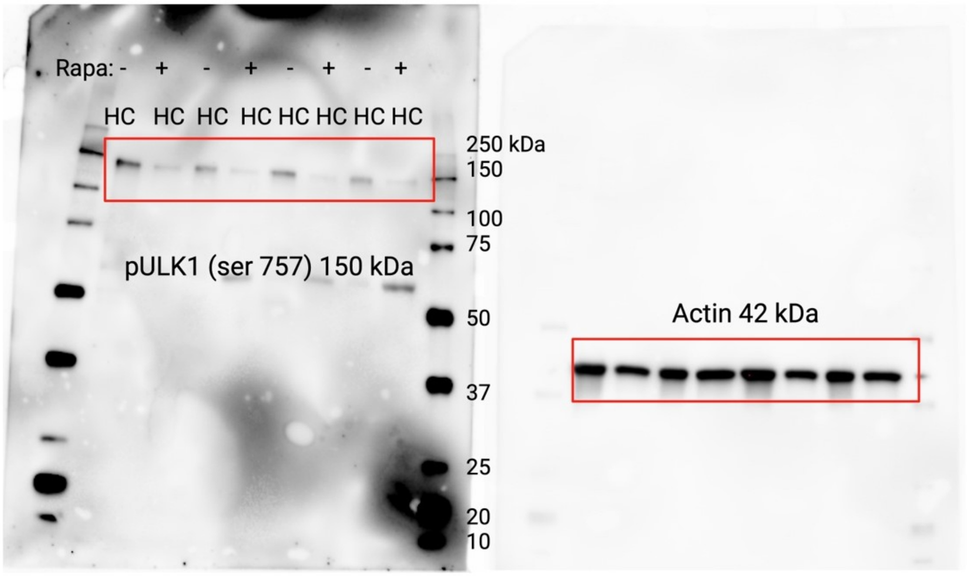
Rapa: - + - + - + - +
HC HC HC HC HC HC HC HC
250 kDa
150
100
75
pULK1 (ser 757) 150 kDa
50
37
25
20
10
Actin 42 kDa

Figure 9H

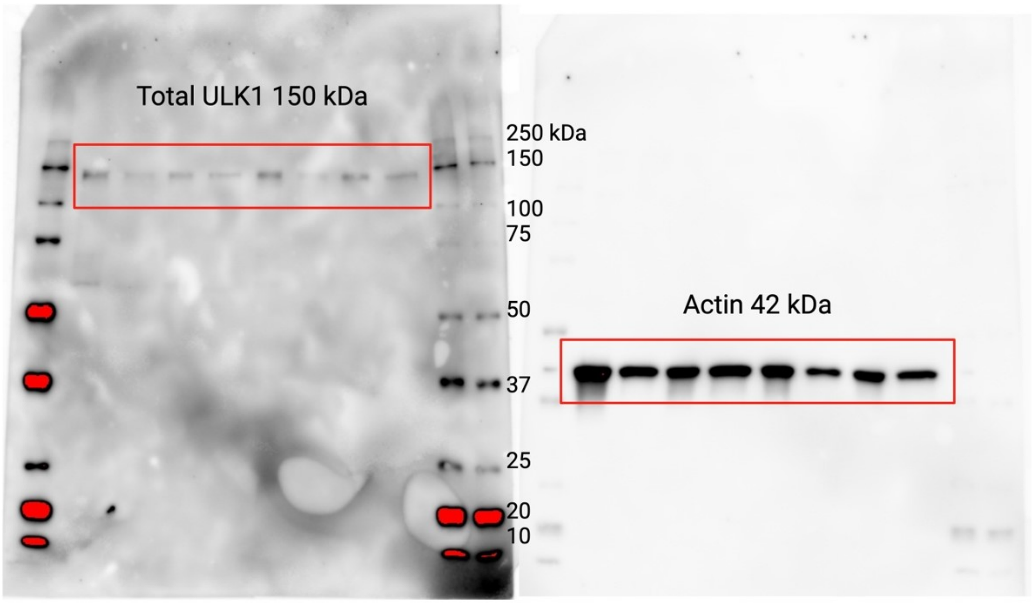
Total ULK1 150 kDa
250 kDa
150
100
75
50
37
25
20
10
Actin 42 kDa

<u>Autophagy Knockout</u>

C2C12 cells treated with or without ATG7 siRNA

Figure 9 I

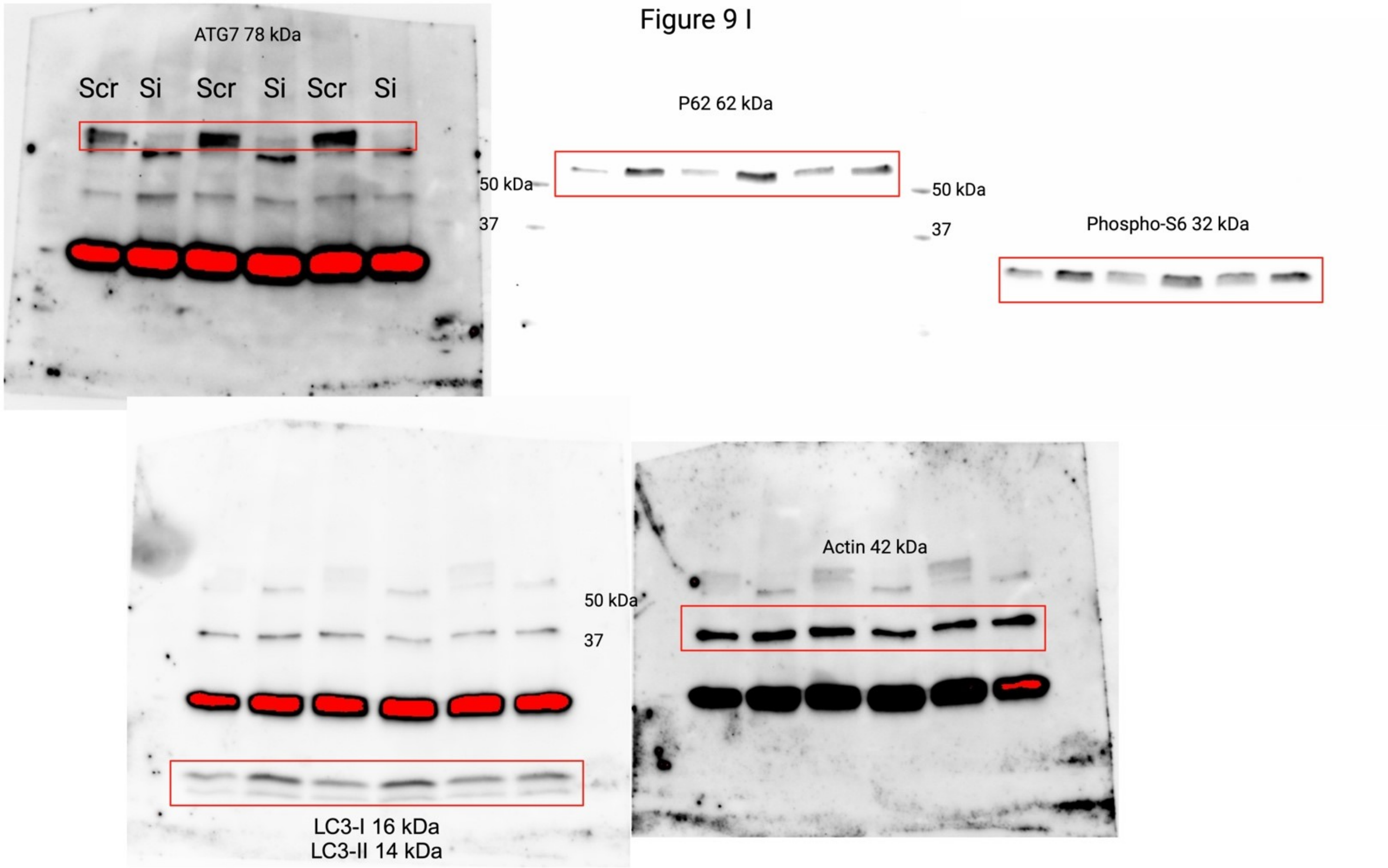

LC3-I/II C2C12 cells treated with Bafilomycin and Rapamycin with or without ATG7 siRNA

Figure 9J

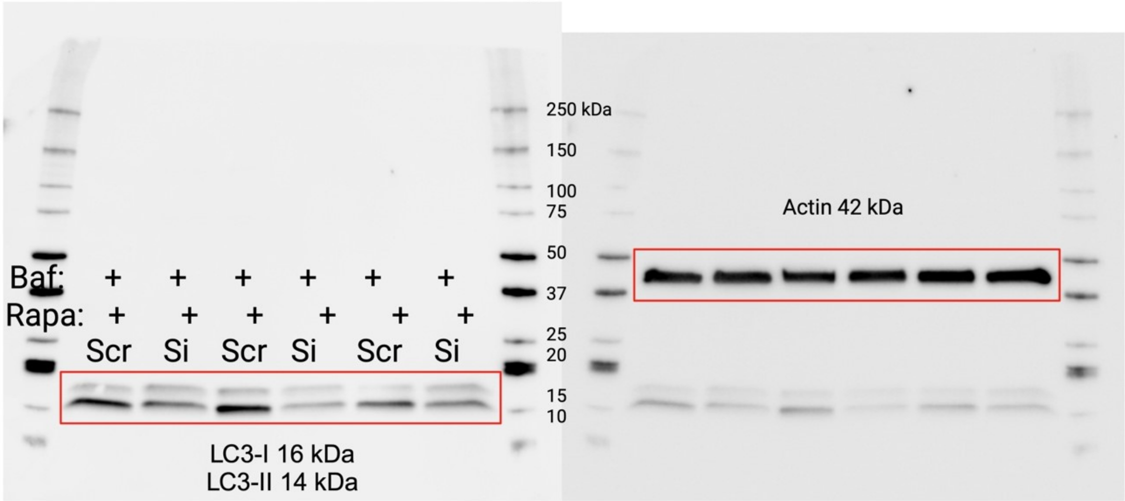

Figure S4

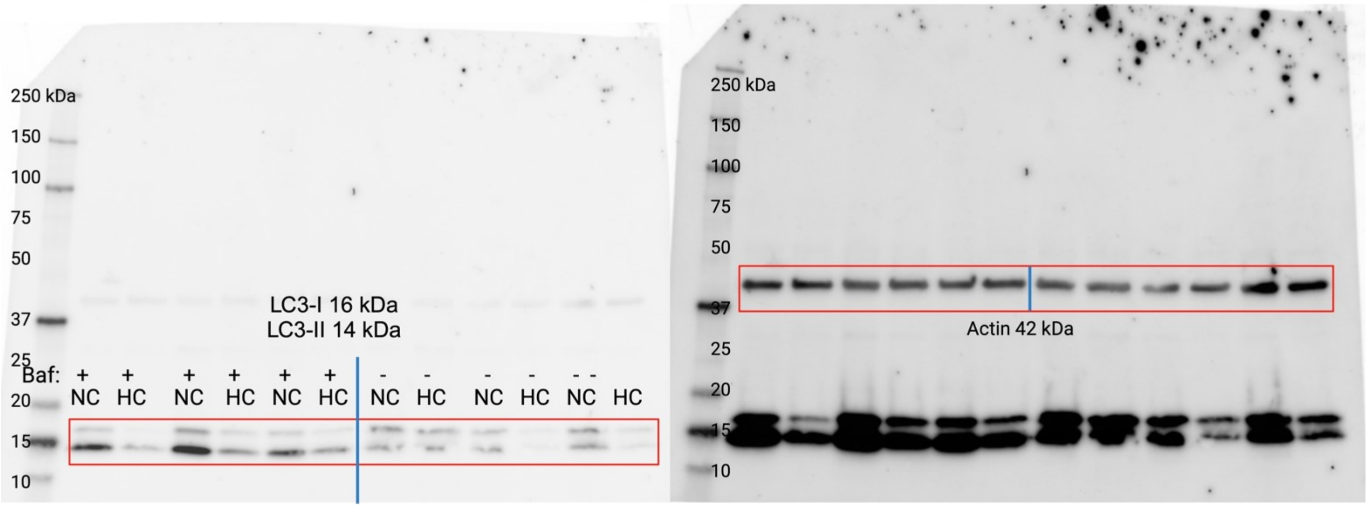

Figure S5

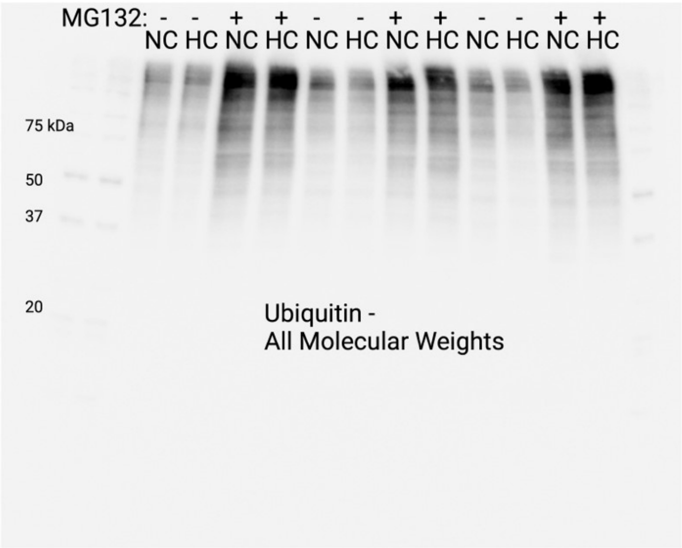

Stain Free AMPKα1 Figure S5 Immunoblot AMPKα1

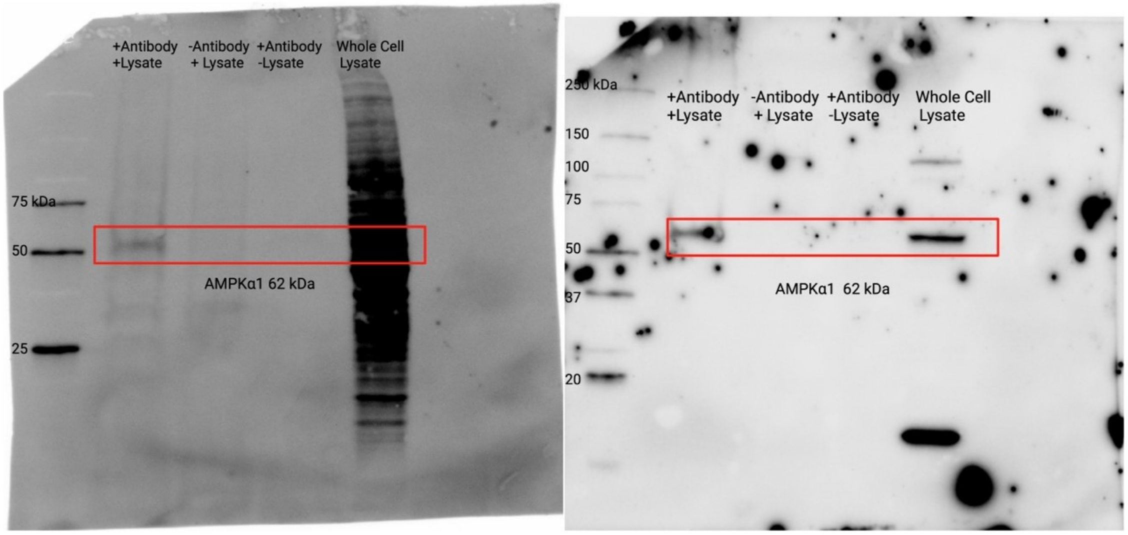

Figure S5 Stain Free AMPKα1

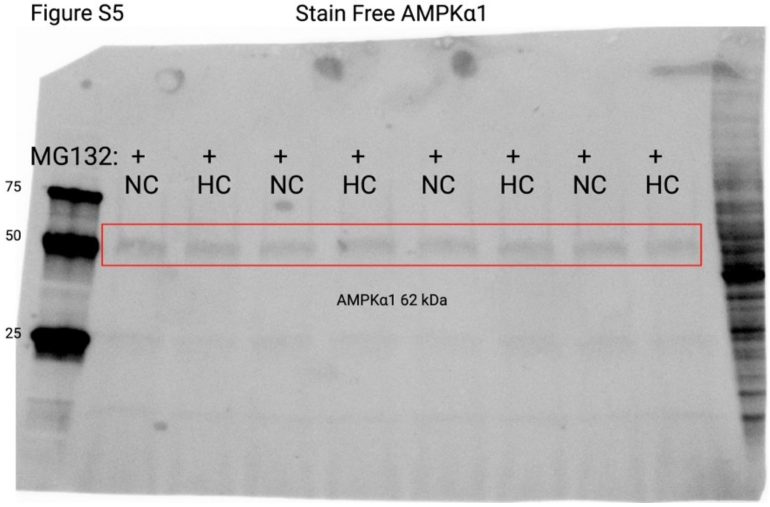

Figure S5

Probed with Ubiquitin

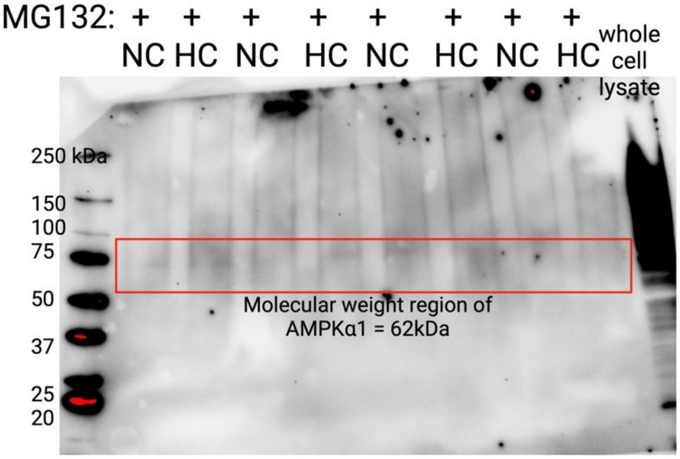

Figure S5

Probed with Total AMPKα1

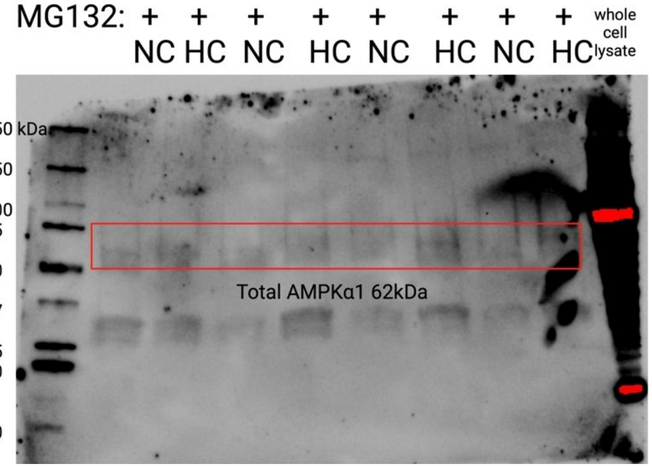

Figure S7

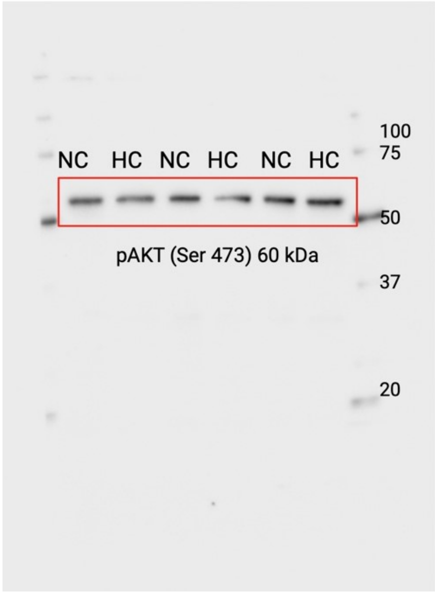

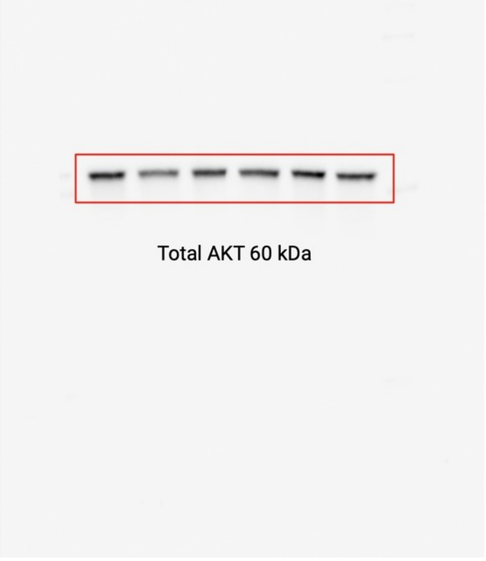

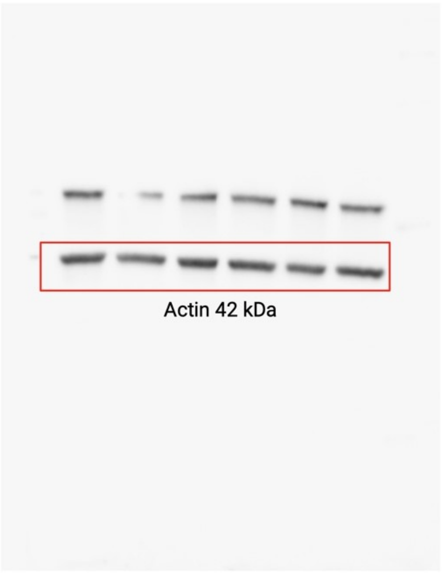

Figure S8

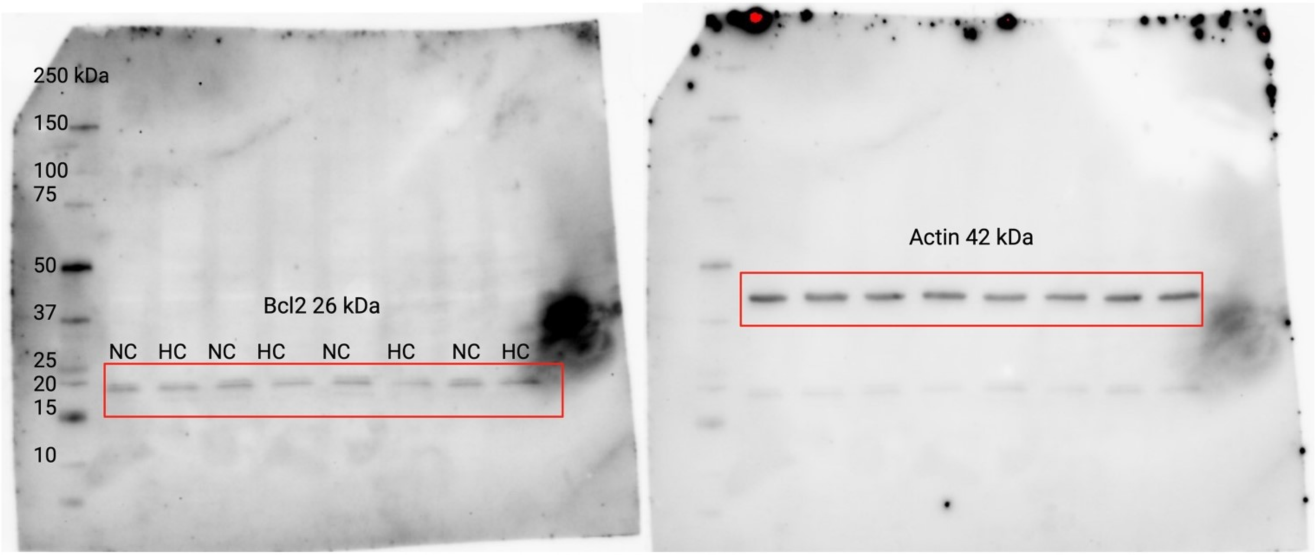

Figure S8

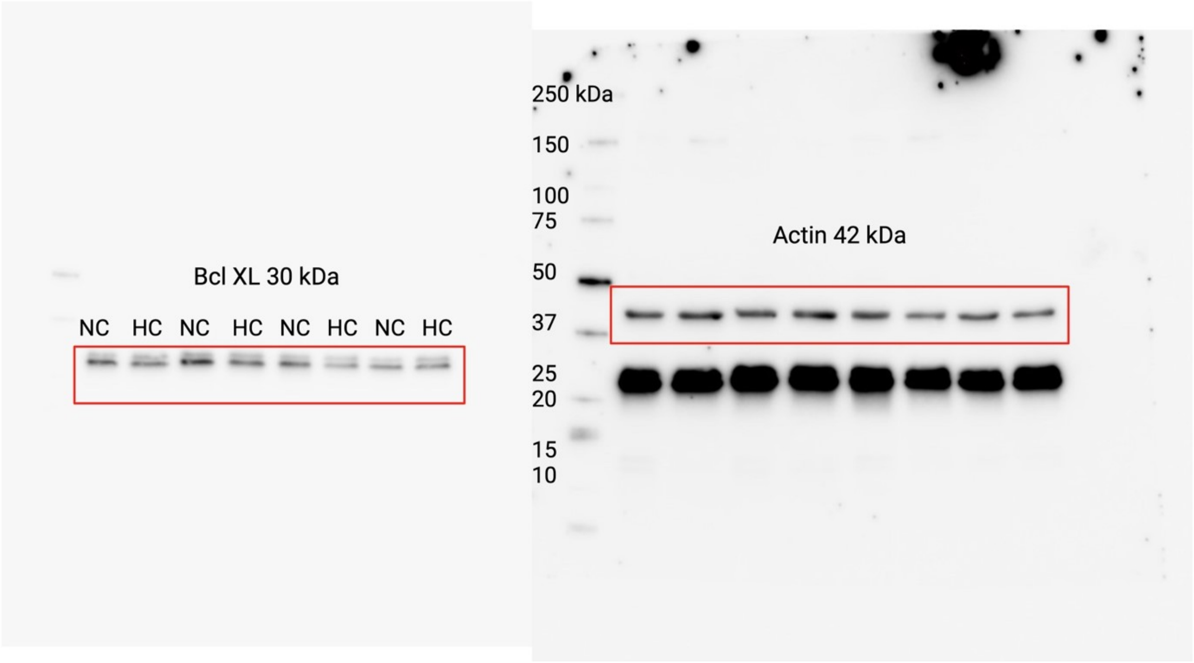

Supplement: Unedited blot and gel images [file jciinsight-10-182842-s062.pdf]
